# Redirecting the Cyanobacterial Bicarbonate Transporters BicA and SbtA to the Chloroplast Envelope: Soluble and Membrane Cargos Need Different Chloroplast Targeting Signals in Plants

**DOI:** 10.3389/fpls.2016.00185

**Published:** 2016-02-29

**Authors:** Vivien Rolland, Murray R. Badger, G. Dean Price

**Affiliations:** Australian Research Council Centre of Excellence for Translational Photosynthesis, Plant Science Division, Research School of Biology, The Australian National University, CanberraACT, Australia.

**Keywords:** chloroplast, bicarbonate transporter, targeting, cyanobacteria, envelope, membrane, transit peptide, photosynthesis

## Abstract

Most major crops used for human consumption are C_3_ plants, which yields are limited by photosynthetic inefficiency. To circumvent this, it has been proposed to implement the cyanobacterial CO_2_-concentrating mechanism (CCM), principally consisting of bicarbonate transporters and carboxysomes, into plant chloroplasts. As it is currently not possible to recover homoplasmic transplastomic monocots, foreign genes must be introduced in these plants *via* nuclear transformation. Consequently, it is paramount to ensure that resulting proteins reach the appropriate sub-cellular compartment, which for cyanobacterial transporters BicA and SbtA, is the chloroplast inner-envelope membrane (IEM). At present, targeting signals to redirect large transmembrane proteins from non-chloroplastic organisms to plant chloroplast envelopes are unknown. The goal of this study was to identify such signals, using *agrobacteria*-mediated transient expression and confocal microscopy to determine the sub-cellular localization of ∼37 GFP-tagged chimeras. Initially, fragments of chloroplast proteins known to target soluble cargos to the stroma were tested for their ability to redirect BicA, but they proved ineffective. Next, different N-terminal regions from *Arabidopsis* IEM transporters were tested. We demonstrated that the N-terminus of *At*HP59, *At*PLGG1 or *At*NTT1 (92–115 amino acids), containing a cleavable chloroplast transit peptide (cTP) and a membrane protein leader (MPL), was sufficient to redirect BicA or SbtA to the chloroplast envelope. This constitutes the first evidence that nuclear-encoded transmembrane proteins from non-chloroplastic organisms can be targeted to the envelope of plant chloroplasts; a finding which represents an important advance in chloroplast engineering by opening up the door to further manipulation of the chloroplastic envelope.

## Introduction

In the last decade, improving plant traits through genetic engineering has become an area of increased focus (Mittler and Blumwald, 2010; Ort et al., 2015). In the context of global food security, improving crop yield has emerged as a critical issue, and maintaining current population growth is predicted to require a doubling of the total food production by 2050 (Zhu et al., 2010; Price et al., 2013; Long et al., 2015). To tackle this problem, several avenues have been proposed; one of them being the implementation of components of the CO_2_-concentrating mechanism (CCM) from cyanobacteria into crop plants such as rice and wheat to improve their photosynthetic capacity (Price et al., 2011a, 2013; McGrath and Long, 2014).

In cyanobacteria, the CCM largely enables the organism to circumvent the catalytic limitations of the CO_2_-fixing enzyme Ribulose-1,5-Bisphosphate Carboxylase/Oxygenase (RuBisCO), which can also inadvertently fix O_2_ into wasteful products if CO_2_ is not in excess (Whitney et al., 2011). This is particularly the case in C_3_ plants that lack any form of CCM, where O_2_ fixation can account for around 25% of the total flux through RuBisCO (Ludwig and Canvin, 1971). The cyanobacterial CCM achieves a large increase in the CO_2_ concentration around RuBisCO via the use of active uptake of CO_2_ and bicarbonate, and release of elevated CO_2_ in the RuBisCO-containing carboxysome structures (Price et al., 2008; Price, 2011; Rae et al., 2013a,b). Recently, it was suggested that the first step toward implementing the cyanobacterial CCM in the chloroplast of key C_3_ crop plants be the addition of bicarbonate transporters into the inner-envelope membrane (IEM) (Price et al., 2013; McGrath and Long, 2014). In cyanobacteria, there are three known bicarbonate transporters: the multi-protein complex BCT1 and the two Na^+^-dependent transporters BicA and SbtA (Omata et al., 1999; Shibata et al., 2002; Price et al., 2004, 2008). Because BicA and SbtA are coded by single genes, they are the more ideal candidates for transfer into higher plants.

Stable expression of cyanobacterial transporters in the chloroplast envelope of higher plants can theoretically be achieved by two alternative methods; transforming the plastid or the nuclear genomes. The main advantage of transforming the plastid genome resides in that it does not require the fusion of chloroplast targeting signals to the proteins of interest. In fact, this method has previously been used to introduce BicA in Tobacco (*Nicotiana tabacum*) chloroplasts (Pengelly et al., 2014). However, when expressed from the plastid genome, BicA had no measurable effect on photosynthesis, possibly because the transporter was not correctly activated (Pengelly et al., 2014). While BicA was also not correctly activated when expressed in *Escherichia coli*, SbtA was functional in this system suggesting that it could also be active in plant chloroplasts (Du et al., 2014).

When aiming at improving crop yield via manipulation of chloroplast physiology, the main limitation of plastid transformation is the current inability to generate homoplasmic transplastomic monocot plants (Hanson et al., 2013). Nuclear transformation therefore appears as the method of choice to introduce BicA and SbtA in higher plant chloroplasts. The main limitation of using this route is that nuclear-encoded heterologous proteins need to be efficiently targeted to the chloroplast envelope in order to increase the C_i_ concentration in the stroma. However, to date, there is a lack of knowledge of how to consistently target foreign membrane proteins to the chloroplast envelope.

Most nuclear-encoded proteins targeted to the chloroplast stroma, IEM or thylakoids, are imported via two translocation multi-protein complexes, TIC and TOC, present on both the outer-envelope membrane (OEM) and the IEM, respectively (see Li and Chiu, 2010). TIC/TOC-mediated import relies on the presence of an N-terminal transit peptide (TP). Upon entry in the chloroplast, part of, or all this signal is cleaved-off by the stromal processing peptidase (SPP) (Richter et al., 2005). The term cTP, for chloroplast transit peptide, has been used previously to represent: (1) the N-terminal protein fragment which is cleaved off in the stroma, and (2) the N-terminal protein domain which is sufficient for efficient chloroplast targeting. To avoid confusion, here we use the term cTP to represent the cleavable peptide, while TP refers to the domain sufficient to target a protein cargo to the chloroplast. This implies that the TP can be equal to the cTP, or longer than the cTP if it also contains part of the mature protein.

In cyanobacteria, BicA and SbtA are located in the plasma membrane and topology studies have revealed that the sequence N-terminal to the first transmembrane domain (TMD) of PCC7002 BicA and PCC7942 SbtA is short, being composed of only 15 and 11 amino acids (aa), respectively (Shelden et al., 2010; Price et al., 2011b). Unsurprisingly, the prediction algorithm ChloroP predicts that BicA and SbtA do not possess a cTP, and as such are not expected to localize in chloroplasts, when nuclear-encoded (Emanuelsson et al., 1999). It is often assumed that the addition of a well characterized cTP such as that of RuBisCO small subunit (RBCS) could target foreign proteins to the chloroplast (Dobberstein et al., 1977; Chua and Schmidt, 1978; Highfield and Ellis, 1978). However, past studies have shown that efficient targeting of foreign proteins requires part of the mature RBCS, in addition to its cTP (Comai et al., 1988; Bionda et al., 2010). Additionally, sequence requirements for chloroplast import of non-plant proteins have only been investigated for soluble proteins targeted to the stroma (Comai et al., 1988; Bionda et al., 2010). Our current understanding of IEM-targeting has been largely shaped by the study of single-pass (i.e., with a single TMD) transmembrane plant proteins such as TIC40, APG1 or ARC6 (Li and Schnell, 2006; Tripp et al., 2007; Viana et al., 2010; Froehlich and Keegstra, 2011). Notably, sequences sufficient to achieve chloroplast envelope targeting of nuclear-encoded multi-pass (i.e., with several TMDs) transmembrane proteins from non-plant organisms have not yet been identified.

In the present study, we set-out to identify appropriate chloroplast-targeting sequences for redirecting BicA and SbtA, using a two-step approach. First, sequences known to efficiently target foreign soluble proteins to the chloroplast stroma were tested for their ability to locate BicA and SbtA to the chloroplast envelope. However, this was shown to be ineffective. BicA (59.6 kD) and SbtA (39.5 kD) are large proteins with 14 and 10 TMDs, respectively (Price and Howitt, 2014). So in a second approach, we turned to analyzing the targeting signals of plant IEM multi-pass proteins. Our analysis shows that these proteins have a long N-terminal domain predicted to be in the stroma, and of about 90–120 aa in length, which is considerably longer than their predicted cTP. The part of these fragments located between the cTP and the first TMD was named membrane protein leader (MPL) and it was speculated that both the cTP and MPL are important for envelope targeting. As a result, the ability of several “cTP+MPL” sequences to target BicA and SbtA to the chloroplast envelope was investigated. Using transient expression of fluorescently tagged constructs, we showed that these cTP+MPL regions were effective, to various degrees, in targeting BicA and SbtA to the chloroplast envelope.

## Materials and Methods

### Prediction of Subcellular Localization, cTP Length, Transmembrane Domains, and Protein Alignments

The subcellular localization of proteins was predicted with TargetP v1.1 (^1^Emanuelsson et al., 2007) and putative cTPs were predicted with Chloro_P v1.1 (^2^Emanuelsson et al., 1999). The TMDs of *At*PLGG1, *At*HP59 and *At*NTT1 were determined using SCAMPI-msa (Bernsel et al., 2008) available on the TOPCONS website (^3^Tsirigos et al., 2015). The list of IEM proteins with at least 10 TMDs was generated using the AT_CHLORO database (^4^Ferro et al., 2010). Protein alignments were generated using ClustalW in Geneious R7^®^.

### Cloning Procedures

The full or partial coding sequence of *A. thaliana TIC20-II* (AT2G47840), *A. thaliana HP59* (AT5G59250), *A. thaliana PLGG1* (AT1G32080), *A. thaliana NTT1* (AT1G80300), *A. thaliana SULTR3;1* (AT3G51895), *Nicotiana benthamiana SULTR3;1* (Nbv5tr6207009), *Pisum sativum RBCS-3C* (X00806), *Glycine max RBCS* (AF303939), *Synechococcus elongatus* PCC7942 SbtA (SYNPCC7942_1475), *Synechococcus sp.* PCC7002 BicA (SYNPCC7002_A2371) and P19 from the Tomato bushy stunt virus (NP_062901), were used in this study. The coding sequence of P19 (gift from Spencer Whitney) was amplified by PCR and inserted in pENTR^®^ (Invitrogen) before being recombined in pMDC32 (Curtis and Grossniklaus, 2003). All other constructs were generated, using or combining sequences synthesized with or without a fluorescent tag, in pUC57, by GENEWIZ, Inc. For detailed information about cloning steps for each construct, see Supplementary Figure S1 (schematic of *AtHP59^93–^BicA-mGFP6::6xHIS::MYC* in pUC57) and Supplementary Tables S1–S3 (Cloning steps for each construct, primer sequence, list of all leaders fused to BicA, SbtA or mGFP6 in this study). Briefly, all constructs were tagged with *mGFP6-6xHIS-MYC* (Haseloff, 1999) or *mTURQUOISE2-6xHIS-MYC* (Goedhart et al., 2012) and recombined in pMDC32 by any of the three following methods: (1) coding sequences were synthesized with a fluorescent tag, and were surrounded by attB sites such that the whole sequence could directly be recombined in pMDC32, (2) coding sequences were synthesized with a fluorescent tag, and had attB sites added through a PCR step, PCR products were recombined in a pDNOR^®^ vector (Invitrogen), which was in turn recombined in pMDC32, (3) coding sequences were synthesized without a tag, and inserted in an attB- and fluorescent tag-containing plasmid generated by methods (1) and (3), prior to being recombined in pMDC32.

### Subcellular Compartment Markers

The plasmids CD3-967 and CD3-959 were used to highlight the Golgi and the ER, respectively (Nelson et al., 2007). These constructs were obtained from the *Arabidopsis* Biological Resource Center (ABRC^5^).

### Plant Materials and Plant Growth

*Nicotiana benthamiana* plants were grown for 3–4 weeks in a CONVIRON growth chamber under a 16 h/8 h day/night cycle with temperatures of 25°C/20°C, a humidity level of 60% and a light intensity of 350–400 μmol photons m^–2^ s^–1^.

### *Agrobacterium* Growth Conditions

*Agrobacterium tumefaciens* GV3101(pMP90) (Koncz and Schell, 1986) were transformed with plasmids of interest and grown in LB media (5 g yeast extract, 10 g tryptone, 10 g NaCl, in 1 l) containing rifampicin (50 μg/ml) and kanamycin (30 μg/ml). Cultures were grown for about 24 h in a 28–30°C incubator and used for transformation of *N. benthamiana* leaves.

### Agro-Infiltration of *Nicotiana benthamiana* Leaves

Bacteria containing P19 (OD600 = 0.3) were mixed with bacteria containing the plasmid of interest and/or the plasmid encoding a subcellular marker (OD600 = 0.5 in each case). Cells were centrifuged for 8 min at 4500 rpm and resuspended in resuspension solution (10 mM 2-[*N*-morpholino]ethanesulfonic acid [MES] pH 5.6, 10 mM MgCl_2_, and 150 μM acetosyringone). The cells were incubated for 2 h at room temperature and infiltrated into 3–4 weeks old *N. benthamiana* leaves. This protocol is adapted from (Breuers et al., 2012).

### Protoplast Preparation

Two days post infiltration (2 dpi), a 4 cm × 4 cm area of infiltrated leaf was cut with a scalpel and transferred in a 5 ml syringe in which 2 ml of digestion solution (1.5% [w/v] cellulase R-10, 0.4% [w/v] macerozyme R-10, 0.4 M mannitol, 20 mM KCl, 20 mM MES pH 5.6, 10 mM CaCl_2_, 0.1% [w/v] BSA) was added and a gentle vacuum was manually applied to facilitated entry of the solution into the intercellular space. The solution and the leaf pieces were transferred to a 2 ml Eppendorf tube and the mixture was incubated 1 h at room temperature. The protoplasts were gently extracted by manually inverting the tube. Leaf debris were removed using a forceps and protoplasts were allowed to sediment before the solution was replaced with imaging solution (0.4 M mannitol, 20 mM KCl, 20 mM MES pH 5.6, 10 mM CaCl2, 0.1% [w/v] BSA). This protocol is adapted from (Breuers et al., 2012).

### Confocal Laser-Scanning Microscopy and Mitochondria Staining

In 2–6 independent experiments, about 100 protoplasts (per independent experiment) expressing GFP-tagged constructs were observed, and several were imaged, using an upright Zeiss LSM780 confocal laser-scanning microscope (Carl Zeiss), a 40x water immersion objective (NA = 1.1) and the Zen 2011 software package (Carl Zeiss). GFP and chlorophyll were excited at 488 nm and emission was recorded at 499–535 nm and at 630–735 nm, respectively. When mCherry was used, it was excited at 561 nm and emission was recorded at 579–633 nm in a separate track. When mTurquoise2 was used, it was excited at 405 nm and emission was recorded at 455–472 nm in a separate track. Mitochondria were stained for 10–20 min using a 100 nM solution of MitoTracker^®^ Red CMXRos (ThermoFisher Scientific). MitoTracker^®^ Red CMXRos was excited at 561 nm and emission was recorded at 570–624 nm.

### Protein Extraction and Western-Blot Analysis

Membrane-enriched protein extracts were prepared from two *N. benthamiana* 2 days post infiltration (dpi) leaf disks of 1.327 cm^2^ each, essentially as follows (Britta Förtser, personal communication). Proteins were extracted from leaf disks by manual grinding in ice–cold extraction buffer (125 mM Tris-HCl pH 8, 1 mM MgCl_2_, 1 mM ethylene-diaminetetraacetic acid [EDTA], 1% [w/v] Polyvinylpolypyrrolidone [PVPP] and 2% [v/v] protease inhibitor cocktail [Sigma]). Debris was removed from the extract after a quick centrifugation step (13000 rpm in a table-top centrifuge, 25°C, 10 s) and membranes were then pelleted with a long centrifugation step (13000 rpm in a table-top centrifuge, 4°C, 20 min). Proteins were extracted from these pellets with resuspension buffer (125 mM Tris-HCl pH 8, 4% (w/v) SDS, 1 mM EDTA) and left overnight at 4°C to allow for full resuspension of the pellets. Proteins samples were separated by SDS-PAGE and transferred to polyvinylidene difluoride (PVDF) membranes. Separate membranes were probed with two different primary antibodies raised in rabbits: anti-GFP (AB6556, Sapphire Bioscience, 1/2000) and anti-TIC40 (AS10709, Agrisera, 1/2500). Blotted membranes were probed with an alkaline phosphatase-conjugated anti-rabbit secondary antibody (BioRad), and the immunoreactive bands were detected using Attophos (Promega) and a VersaDoc imager (Biorad).

## Results

### Nuclear-Encoded Cyanobacterial BicA and SbtA are Not Targeted to Chloroplasts in *Nicotiana benthamiana*

**FIGURE 1:**
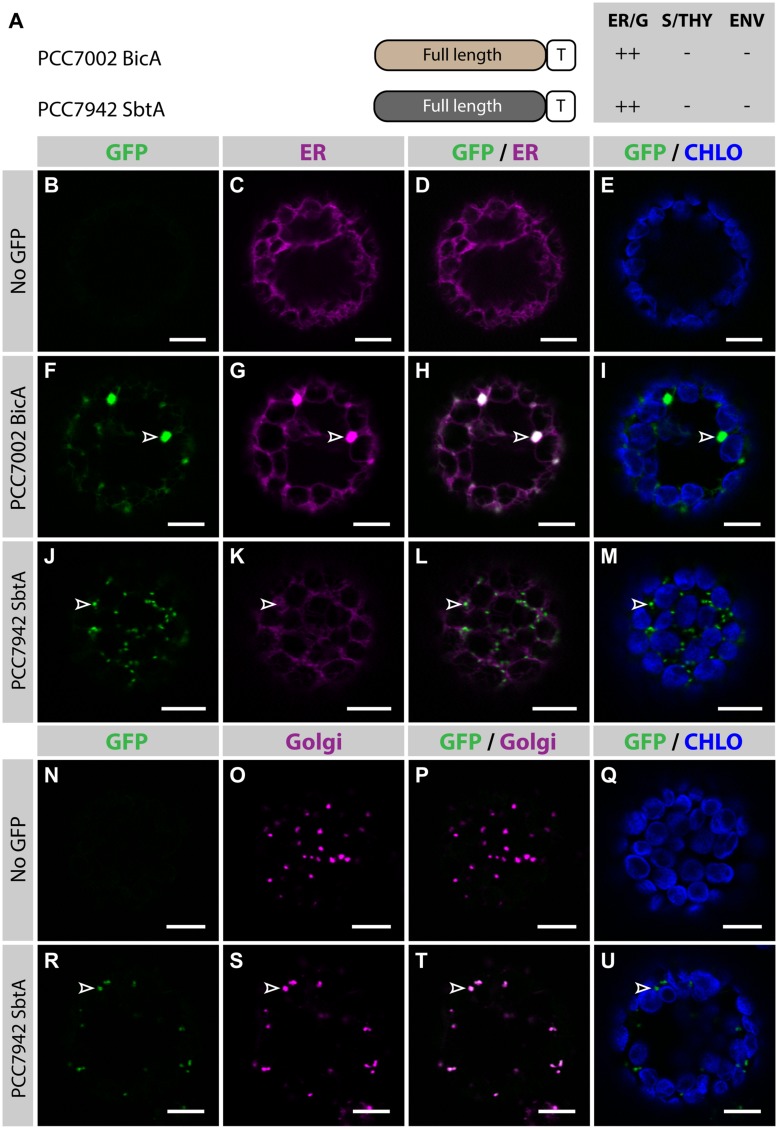
**Nuclear-encoded BicA and SbtA do not localize in chloroplasts in *Nicotiana benthamiana.* (A)** Schematic of PCC7002 BicA and PCC7942 SbtA, and summary of their subcellular distribution averaged from a total of at least 50 protoplasts. For each chimera, the compartment where most of a protein was found (primary targeting) was indicated as ++, while secondary targeting, traces, and no detectable signal were indicated as +, +/−, and −, respectively. ER/G, endoplasmic reticulum or Golgi apparatus; S/THY, stroma or thylakoids; ENV, chloroplast envelope; T, GFP-containing tag. **(B–U)**, Single-plane confocal microscopy images of *N. benthamiana* protoplasts expressing a GFP-tagged chimera **(F,J,R)** or none for controls **(B,N)** together with an ER **(C,G,K)** or Golgi **(O,S)** marker, 2 days post infiltration (dpi). Merges of GFP with ER **(D,H,L)**, Golgi **(P,T)** or chlorophyll signal **(E,I,M,Q,U)** are also shown. These images show that PCC7002 BicA co-localized with the ER marker (empty arrowheads in **F–I**) and that PCC7942 SbtA did not (empty arrowheads in **J–M**). Instead, PCC7942 SbtA co-localizes with the Golgi marker (empty arrowheads in **R–U**). Scales bars: 10 μm.

To determine the subcellular localization of nuclear-encoded PCC7002 BicA and PCC7942 SbtA (hereafter referred to as BicA and SbtA, respectively), constructs containing their coding sequence fused to GFP were transiently expressed in the leaves of *N. benthamiana via* agro-infiltration, and isolated protoplasts were observed using confocal microscopy (**Figure 1**). To enable comparison of chloroplast-targeting efficiency of the different constructs tested in this study, we systematically analyzed protein distribution on protoplasts prepared 2 days post infiltration (dpi); a time-point late enough to allow protein expression and early enough to limit effects of protein over-expression on subcellular localization. As predicted, at 2 dpi, neither BicA nor SbtA were found in chloroplasts. Instead, BicA co-localized with an endoplasmic reticulum (ER) marker (**Figures 1F–I**), while SbtA was only weakly expressed in the ER and accumulated in bright foci throughout the cell (**Figures 1J–M**). The identity of these foci was tested by staining SbtA-expressing protoplasts with a mitochondrial stain or a marker of the Golgi apparatus. This experiment revealed that SbtA was not located in mitochondria (Supplementary Figure S2), but instead localized in the Golgi apparatus (**Figures 1R–U**). These results highlighted the inability of nuclear-encoded BicA and SbtA to reach plastids and prompted us to identify efficient foreign targeting signals to redirect them to chloroplasts.

### The cTP Plus a Part of the Mature RBCS is Necessary and Sufficient to Efficiently Target GFP, but Not BicA, to Chloroplasts

In a previous study, Comai et al. (1988) analyzed the ability of different parts of RBCS to target a foreign soluble cargo, namely the 5-enolpyruvyl-3-phosphoshikimate synthase (EPSP) from *Salmonella typhimurium*, to chloroplasts, both *in vitro* and *in vivo*. They found that the cTP of Soybean (*Glycine max, Gm*) RBCS alone was not enough for import in chloroplasts. However, the addition of the first 25 aa of the mature part of RBCS from Pea (*Pisum sativum, Ps*) was sufficient to target EPSP to chloroplasts both *in vitro* and *in vivo* (Comai et al., 1988).

**FIGURE 2:**
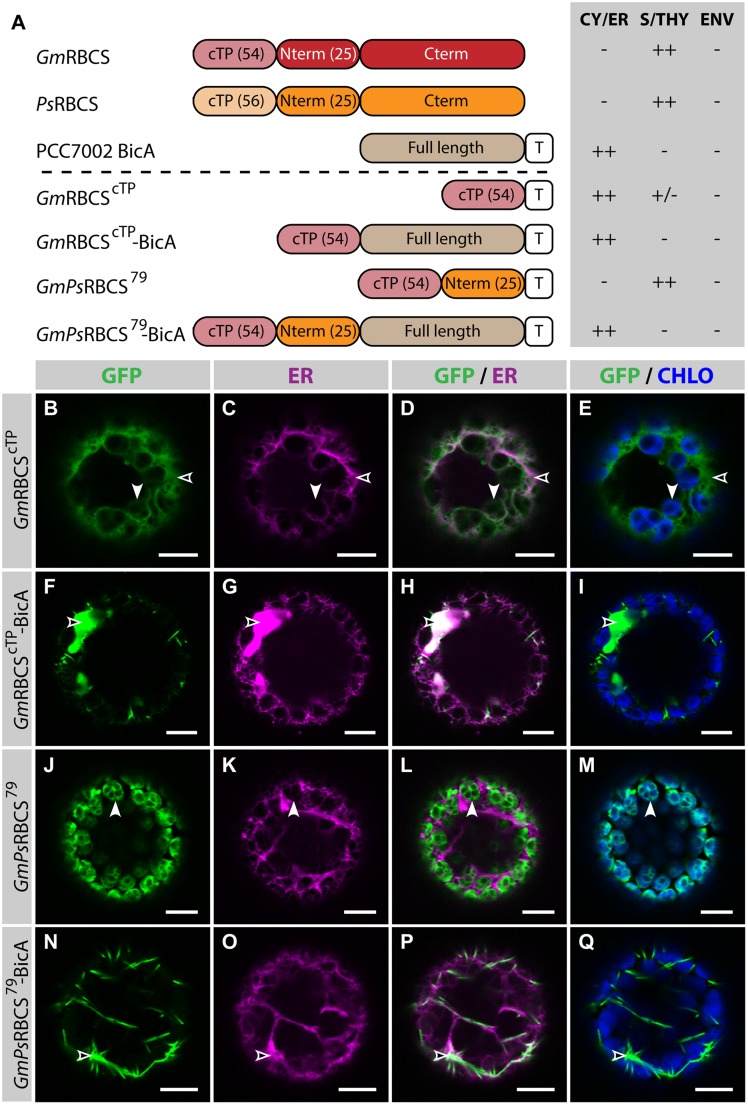
***GmPs*RBCS^79^ can target GFP, but not BicA, to chloroplasts. (A)** Schematic of the RBCS/BicA chimeras used in this figure together with a summary of their subcellular distribution as explained in **Figure 1.** CY/ER, cytosol or endoplasmic reticulum; S/THY, stroma or thylakoids; ENV, chloroplast envelope; T, GFP-containing tag. Numbers in brackets indicate the number of aa making-up protein domains. The subcellular localization of *Gm*RBCS and *Ps*RBCS were inferred from the literature. **(B–Q)** Single-plane confocal microscopy images of *N. benthamiana* protoplasts expressing a GFP-tagged chimera **(B,F,J,N)** together with an ER marker **(C,G,K,O)**, 2 dpi. Merges of GFP with ER **(D,H,L,P)** or chlorophyll signal **(E,I,M,Q)** are also shown. These images show that *Gm*RBCS^cTP^ localized mainly in the cytosol (empty arrowheads in **B–E**) and only weakly to chloroplasts (arrowheads in **B–E**), *Gm*RBCS^cTP^-BicA was found in the ER (empty arrowheads in **F–I**), *GmPs*RBCS^79^ was entirely targeted to chloroplasts (arrowheads in **J–M**) and *GmPs*RBCS^79^-BicA accumulated outside of chloroplasts (empty arrowheads in **N–Q**). Scales bars: 10 μm.

In the present study, similar RBCS fragments were fused to GFP or BicA-GFP, to analyze their ability to direct a soluble and a transmembrane cargo to chloroplasts, respectively (**Figure 2**; Supplementary Figure S3). The cTP of *Gm*RBCS fused to GFP (*Gm*RBCS^cTP^) was mostly distributed in the cytosol, and was only poorly translocated to the chloroplast, with traces only detected in the stroma (**Figures 2B–E**). Similarly, the cTP of *Ps*RBCS (*Ps*RBCS^cTP^) only achieved partial chloroplast targeting, with most of the GFP localized in the cytosol (Supplementary Figures S3B–E). These results are consistent with another study in which confocal microscopy was used to study the TP length required to target a foreign soluble cargo, the 27th Ig domain of the muscle protein TITIN (referred to below as TITIN), to chloroplasts (Bionda et al., 2010). In this study, Bionda et al. (2010) showed that the cTP plus 5 aa of the mature *Nicotiana tabacum* RBCS was only able to achieve efficient targeting of TITIN in 52% of the protoplasts observed; 46.5% of the protoplasts had GFP in both the chloroplast and the cytosol and 1.5% had GFP in the cytosol only. Given that here *Gm*RBCS^cTP^ and *Ps*RBCS^cTP^ were unable to efficiently target GFP to chloroplasts, it was not surprising to observe that *Gm*RBCS^cTP^-BicA was not imported in chloroplasts and instead localized in the ER (**Figures 2F–I**).

When 25 aa of the mature *Ps*RBCS were added to *Gm*RBCS^cTP^ (resulting in *GmPs*RBCS^79^), GFP was efficiently targeted to chloroplasts (**Figures 2J–M**), consistent with previous observations (Comai et al., 1988). In contrast to this result, both *GmPs*RBCS^79^-BicA and *Gm*RBCS^79^-BicA, a construct in which the first 79 aa of *Gm*RBCS were fused to BicA, were unable to redirect BicA to chloroplasts (**Figures 2N–Q**; Supplementary Figures S3F–I). Taken together, these results highlighted that sequences required for chloroplast-targeting of a soluble or a large transmembrane protein are different.

### Fragments of *At*NTT1 Which can Efficiently Target GFP to Chloroplasts Fail to Redirect BicA to Chloroplasts

The Nucleotide Transporter 1 from *Arabidopsis thaliana* (*At*NTT1) is a nuclear-encoded transmembrane protein, which localizes in the IEM of chloroplasts (Neuhaus et al., 1997). Bionda et al. (2010) analyzed the subcellular distribution of different *At*NTT1-TITIN chimeric fusions and found that the cTP of NTT1 alone (*At*NTT1^cTP^, 21 aa), or combined with the first 9, 19, or 29 aa of mature *At*NTT1 (*At*NTT1^30^, *At*NTT1^40^ and *At*NTT1^50^, respectively), were unable to target TITIN to chloroplasts. However, when the *At*NTT1 N-terminal fragment used was extended by another 10 or 20 aa (*At*NTT1^60^ and *At*NTT1^70^, respectively), the chimeras were almost completely targeted to chloroplasts (Bionda et al., 2010). These results showed that the first 60 aa of *At*NTT1 are sufficient for near complete chloroplast targeting of a soluble cargo.

**FIGURE 3:**
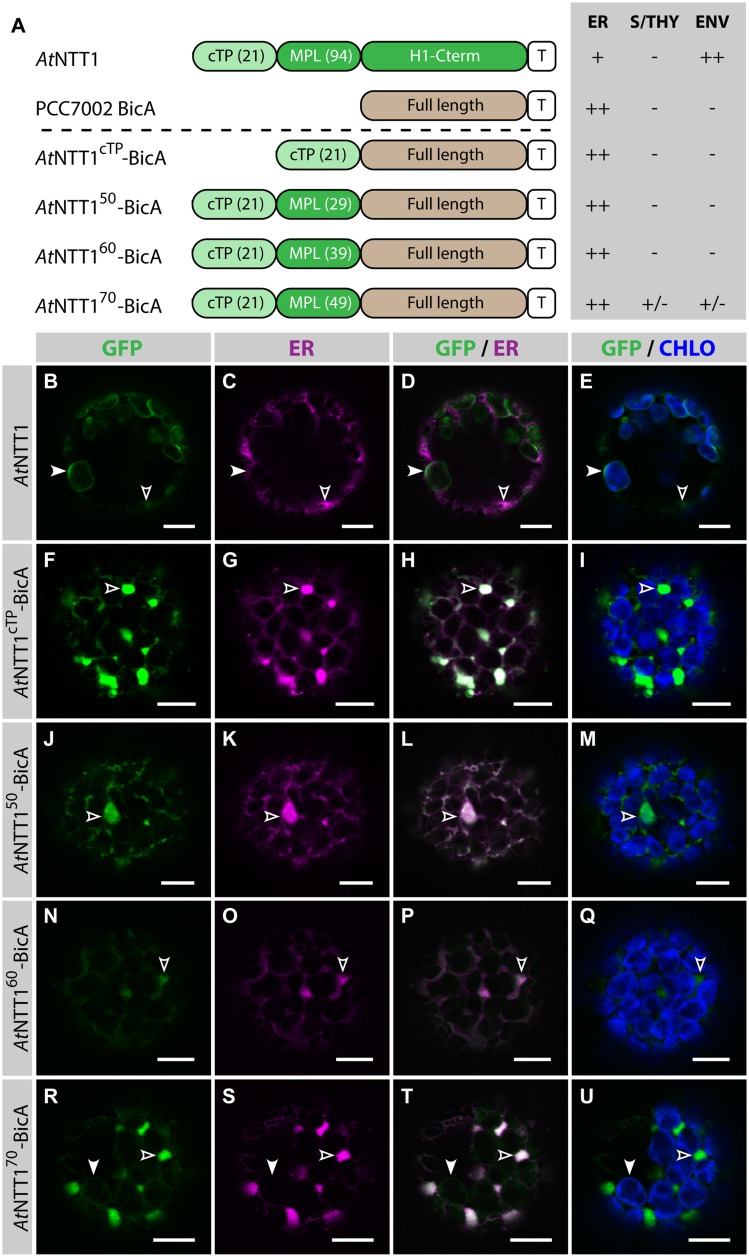
**The first 70 aa of *At*NTT1 fail to efficiently deliver BicA to chloroplasts. (A)** Schematic of the *At*NTT1/BicA chimeras used in this figure together with a summary of their subcellular distribution as explained in **Figure 1.** ER, endoplasmic reticulum; S/THY, stroma or thylakoids; ENV, chloroplast envelope; T, GFP-containing tag. Numbers in brackets indicate the number of aa making-up protein domains. **(B–U)** Single-plane confocal microscopy images of *N. benthamiana* protoplasts expressing a GFP-tagged chimera **(B,F,J,N,R)** together with an ER marker **(C,G,K,O,S)**, 2 dpi. Merges of GFP with ER **(D,H,L,P,T)** or chlorophyll signal **(E,I,M,Q,U)** are also shown. These images show that *At*NTT1 **(B–E)** mainly localized in the chloroplast envelope (arrowheads) with only weak signal in the ER (empty arrowheads), that *At*NTT1^cTP^-BicA **(F–I)**, *At*NTT1^50^-BicA **(J–M)** and *At*NTT1^60^-BicA **(N–Q)** were entirely localized in the ER (empty arrowheads), and that *At*NTT1^70^-BicA **(R–U)** was mainly localized in the ER (empty arrowheads) with only traces detected in chloroplasts (arrowheads). Scales bars: 10 μm.

To test whether the same length was sufficient to direct a transmembrane cargo to plastids, we fused *At*NTT1^cTP^, *At*NTT1^50^, *At*NTT1^60^, and *At*NTT1^70^ to BicA-GFP and analyzed the subcellular localization of the resulting chimeras (**Figure 3**). As expected, GFP-tagged *At*NTT1 localized mainly in the chloroplast envelope (**Figures 3B–E**), with little signal detected in the ER, while *At*NTT1^cTP^-BicA and *At*NTT1^50^-BicA localized to the ER only (**Figures 3F–M**). Notably, *At*NTT1^60^-BicA was found in the ER (**Figures 3N–Q**), while *At*NTT1^70^-BicA accumulated mainly in the ER with only traces of signal in the chloroplast (**Figures 3R–U**). These results were different from what Bionda et al. (2010) observed with the soluble TITIN cargo and confirmed our own observation based on the use of RBCS fragments that large transmembrane cargos were harder to redirect than soluble cargos. These results highlighted the importance of identifying transmembrane-specific chloroplast-targeting signals.

### Selection of Plant Protein Candidates with Potential Transmembrane-Specific Chloroplast-Targeting Signals

A potential source of transmembrane-specific targeting sequences could be chloroplast-targeted plant homologues of SbtA or BicA. While SbtA is widespread in cyanobacteria and found in a few bacteria, it does not have homologues in eukaryotes (Price and Howitt, 2014). BicA, however, is part of the SulP/SLC26 family of eukaryotic and prokaryotic transporters (Price et al., 2004; Price and Howitt, 2011). In *A. thaliana*, the members of this family are known as Sulphate Transporters (SULTR) (Price and Howitt, 2011). Interestingly, *At*SULTR3;1 was recently reported to localize in chloroplasts (Cao et al., 2013). One of the main differences between *At*SULTR3;1 and BicA is the length of their stromal/cytoplasmic N-terminus, which is significantly longer in *At*SULTR3;1 (85 aa) than in BicA (15 aa). Albeit long, the N-terminus of *At*SULTR3;1 is not expected to contain a cTP, according to ChloroP predictions. The subcellular localization of *At*SULTR3;1-GFP and *Nb*SULTR3;1-GFP was therefore tested, but surprisingly both proteins localized in the ER (Supplementary Figure S4), ruling out the possibility of using parts of SULTR3;1 to direct BicA and SbtA to chloroplasts.

Because homologs of BicA and SbtA could not be used, it became important to identify substitute candidate proteins. It was reasoned that ideal putative candidates should have several TMDs and localize in the IEM. A list of *A. thaliana* IEM-localized proteins was built using the chloroplast proteomics database AT_CHLORO (Ferro et al., 2010). This list was curated to only include IEM proteins which had 10 or more TMDs, and a predicted cTP. The following eight proteins possessed both these attributes: *At*PLGG1, *At*HP59, *At*NTT1, *At*PHT2;1, *At*GLT1, *At*NTT2, *At*DIT2;2 and *At*CLT2. The Glycolate-Glycerate transporter *At*PLGG1 and the putative D-Xylose transporter *At*HP59 were selected for further in-depth analyses (Runquist et al., 2010; Pick et al., 2013).

### Both the cTP and the MPL are Required for Chloroplast-Targeting of *At*HP59 and *At*PLGG1

**FIGURE 4:**
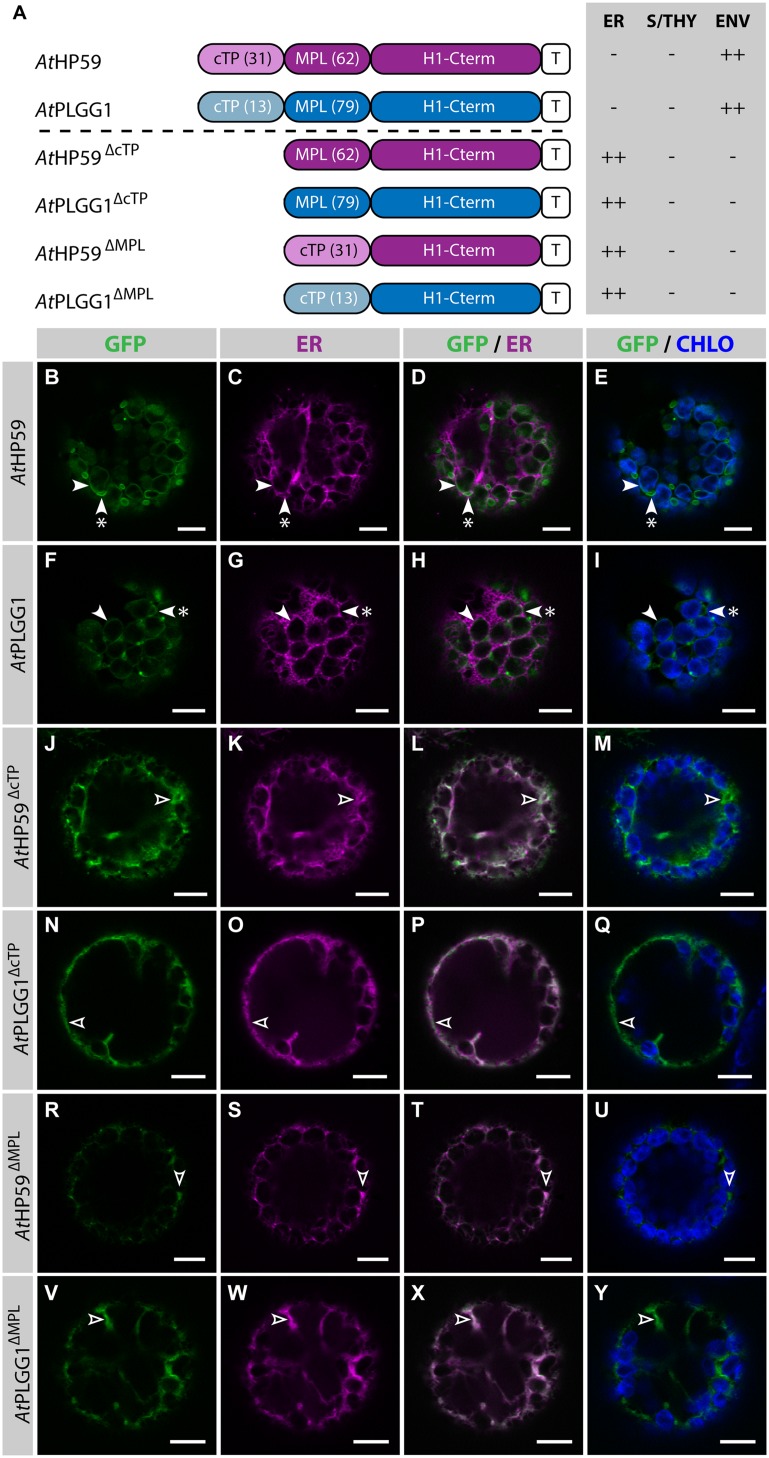
***At*HP59 and *At*PLGG1 require both their cTP and their MPL for chloroplast targeting. (A)** Schematic of the *At*HP59 and *At*PLGG1 chimeras in which the cTP or the MPL was deleted, together with a summary of their subcellular distribution as explained in **Figure 1.** ER: endoplasmic reticulum; S/THY, stroma or thylakoids; ENV, chloroplast envelope; T, GFP-containing tag. Numbers in brackets indicate the number of aa making-up protein domains. **(B–Y)** Single-plane confocal microscopy images of *N. benthamiana* protoplasts expressing a GFP-tagged chimera **(B,F,J,N,R,V)** together with an ER marker **(C,G,K,O,S,W)**, 2 dpi. Merges of GFP with ER **(D,H,L,P,T,X)** or chlorophyll signal **(E,I,M,Q,U,Y)** are also shown. These images show that *At*HP59 **(B–E)** and *At*PLGG1 **(F–I)** were targeted to the chloroplast envelope (arrowheads) where they formed stromules (starred arrowheads). In contrast, *At*HP59^ΔcTP^ **(J-M)**, *At*PLGG1^ΔcTP^ **(N–Q)**, *At*HP59^ΔMPL^ **(R–U)** and *At*PLGG1^ΔMPL^ **(V–Y)** all localized in the ER (empty arrowheads). Scales bars: 10 μm.

Prior to determining whether *At*HP59 and *At*PLGG1 leader sequences may be used to redirect BicA and SbtA, the domains important for their own IEM localization were investigated (**Figure 4**; Supplementary Figure S5). When fused to GFP, both *At*HP59 and *At*PLGG1 localized in the chloroplast envelope, where their expression triggered the formation of stroma-filled envelope protrusions called stromules, consistent in shape with IEM localization (**Figures 4B–I**) (Breuers et al., 2012). N-terminal to their first TMD, *At*HP59 and *At*PLGG1 had a long putative stromal domain of 93 and 92 aa, respectively, which could be subdivided into a cTP and a membrane protein leader (MPL), i.e., the sequence between the cTP and the first TMD. More specifically, the N-terminus of *At*HP59 contained a cTP of 31 aa and a MPL of 62 aa, and that of *At*PLGG1 was made of a cTP of 13 aa and a MPL of 79 aa (**Figure 4A**). To test the requirement of either domain for chloroplast localization, the cTP and the MPL of both proteins were individually deleted. Deleting the cTPs in *At*PLGG1^ΔcTP^ or *At*HP59^ΔcTP^ resulted in failure to reach the chloroplast and accumulation of the proteins in the ER (**Figures 4J–Q**). When deleting the MPLs, in *At*PLGG1^ΔMPL^ and *At*HP59^ΔMPL^, the 5 C-terminal aa of each MPL were retained to avoid possible protein instability due to the perturbation of their first TMD (for aa sequence, see Supplementary Figure S5). Just like *At*PLGG1^ΔcTP^ and *At*HP59^ΔcTP^, *At*PLGG1^ΔMPL^ and *At*HP59^ΔMPL^ localized in the ER (**Figures 4R–Y**). These results indicated that both the cTP and the MPL are essential for chloroplast targeting of *At*PLGG1 or *At*HP59.

**FIGURE 5:**
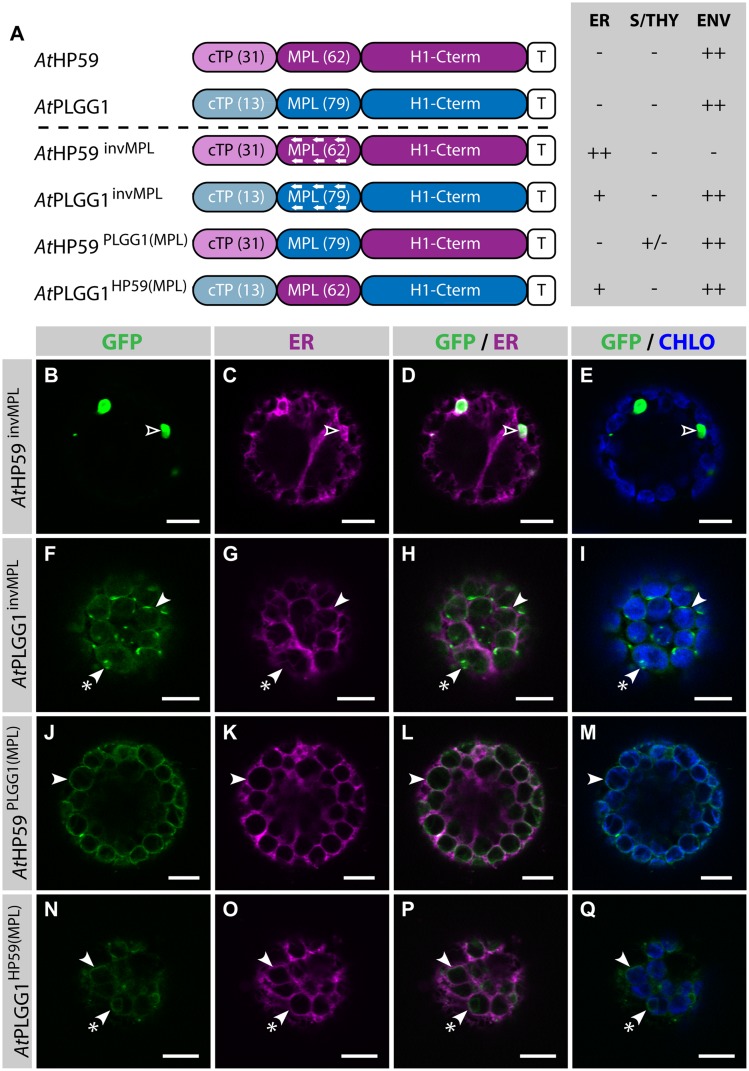
**MPL signals can be orientation-sensitive and need to be combined with the right cTP for efficient chloroplast targeting of *At*HP59 and *At*PLGG1. (A)** Schematic of the *At*HP59 and *At*PLGG1 chimeras in which the MPL sequence was inverted or swapped, together with a summary of their subcellular distribution as explained in **Figure 1.** ER, endoplasmic reticulum; S/THY, stroma or thylakoids; ENV, chloroplast envelope; T, GFP-containing tag. Numbers in brackets indicate the number of aa making-up protein domains. **(B–Q)** Single-plane confocal microscopy images of *N. benthamiana* protoplasts expressing a GFP-tagged chimera **(B,F,J,N)** together with an ER marker **(C,G,K,O)**, 2 dpi. Merges of GFP with ER **(D,H,L,P)** or chlorophyll signal **(E,I,M,Q)** are also shown. These images show that *At*HP59^invMPL^ **(B–E)** localized in vesicles (empty arrowheads) outside of the chloroplasts, while *At*PLGG1^invMPL^ **(F–I)**, *At*HP59^PLGG1(MPL)^ **(J–M)** and *At*PLGG1^HP59(MPL)^ **(N–Q)** localized mainly in the chloroplast envelope (arrowheads), where they formed stromules (starred arrowheads). Scales bars: 10 μm.

Strikingly, in the cTP+MPL of *At*HP59 aa charges are distributed asymmetrically, while this is not the case in the cTP+MPL of *At*PLGG1 (Supplementary Figure S5). To test whether the information contained in the N-terminus of both proteins was orientation-sensitive or could be used in a different context, their MPLs were successively inverted or swapped (**Figure 5**; Supplementary Figure S5). In the process of inversion, the aa sequence of each MPL was reversed, e.g., Asp-Lys-Pro becoming Pro-Lys-Asp, resulting in *At*HP59^invMPL^ and *At*PLGG1^invMPL^. Just as in constructs in which the MPLs were deleted, in *At*HP59^invMPL^ and *At*PLGG1^invMPL^ the 5 C-terminal aa of each MPL were kept in their original orientation to limit sequence perturbation around the first TMD (**Figure 5A**; Supplementary Figure S5). Notably, *At*HP59^invMPL^ did not reach the chloroplast and accumulated in large cytoplasmic vesicles (**Figures 5B–E**). Contrastingly, *At*PLGG1^invMPL^ localized mainly in the chloroplast envelope, where it formed stromules, and only weak signal was detected in the ER (**Figures 5F–I**). The fact that *At*HP59^invMPL^, unlike *At*HP59, *At*PLGG1, and *At*PLGG1^invMPL^, was not able to translocate to chloroplasts might be due to altered charge distribution in its N-terminus (see Supplementary Figure S5 and discussion).

MPL interchangeability was then tested by replacing the MPL of *At*HP59, with that of *At*PLGG1 (resulting in *At*HP59^PLGG1(MPL)^), and *vice versa* (resulting in *At*PLGG1^HP59(MPL)^) (**Figures 5J–Q**; Supplementary Figure S5). Again, in these constructs the 5 C-terminal aa of each endogenous MPL were not modified to limit protein instability. Both MPL-swapped chimeras were targeted to the chloroplast, where they formed stromules suggesting IEM localization. Most of *At*PLGG1^HP59(MPL)^ was found in the chloroplast envelope, with little signal observed in the ER (**Figures 5N–Q**). *At*HP59^PLGG1(MPL)^ was more efficiently targeted to the chloroplast, with no detectable signal in the ER and only traces of protein detected inside chloroplasts (**Figures 5J–M**). These results highlighted that these MPLs could be exchanged and yet maintain chloroplast targeting, although native cTP+MPL combinations resulted in more efficient chloroplast translocation. A possible explanation for why *At*PLGG1^HP59(MPL)^ was partially found in the ER but *At*HP59^PLGG1(MPL)^ was not, might be that the cTP+MPL combination in *At*PLGG1^HP59(MPL)^ was considerably shorter, and possibly weaker, than that of *At*HP59^PLGG1(MPL)^, with 75 aa and 110 aa, respectively (Supplementary Figure S5). These experiments do, however, indicate that the ∼90–115 aa length of the cTP+MPL is a common feature of successful envelope targeting.

### Chloroplast Targeting of BicA and SbtA Using a Combination of cTP+MPL from *At*HP59, *At*PLGG1 or *At*NTT1

**FIGURE 6:**
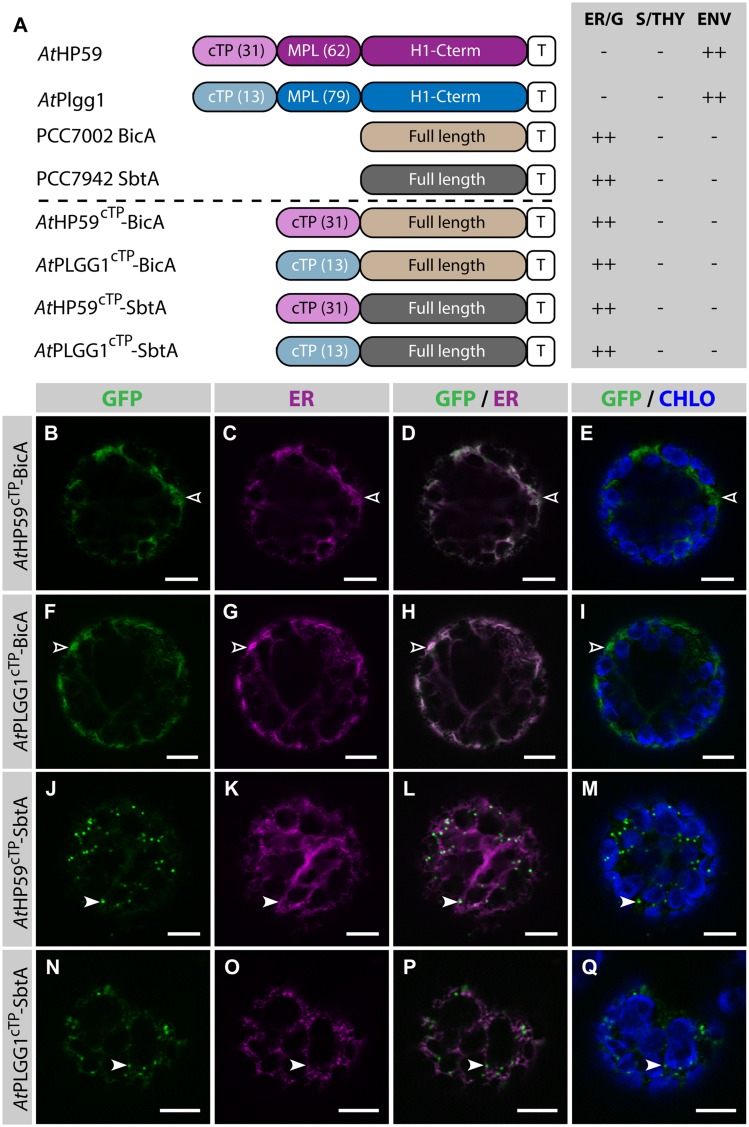
**The cTPs of *At*HP59 and *At*PLGG1 are not sufficient to target BicA and SbtA to chloroplasts. (A)** Schematic of the chimeras used in this figure, together with a summary of their subcellular distribution as explained in **Figure 1.** ER, endoplasmic reticulum; S/THY, stroma or thylakoids; ENV, chloroplast envelope; T, GFP-containing tag. Numbers in brackets indicate the number of aa making-up protein domains. **(B–Q)** Single-plane confocal microscopy images of *N. benthamiana* protoplasts expressing a GFP-tagged chimera **(B,F,J,N)** together with an ER marker **(C,G,K,O)**, 2 dpi. Merges of GFP with ER **(D,H,L,P)** or chlorophyll signal **(E,I,M,Q)** are also shown. These images show that *At*HP59^cTP^-BicA **(B–E)** and *At*PLGG1^cTP^-BicA **(F–I)** localized in the ER (empty arrowheads), while *At*HP59^cTP^-SbtA **(J–M)** and *At*PLGG1^cTP^-SbtA **(N–Q)** were mainly found in the Golgi (arrowheads), with traces of signal in the ER. Scales bars: 10 μm.

**FIGURE 7:**
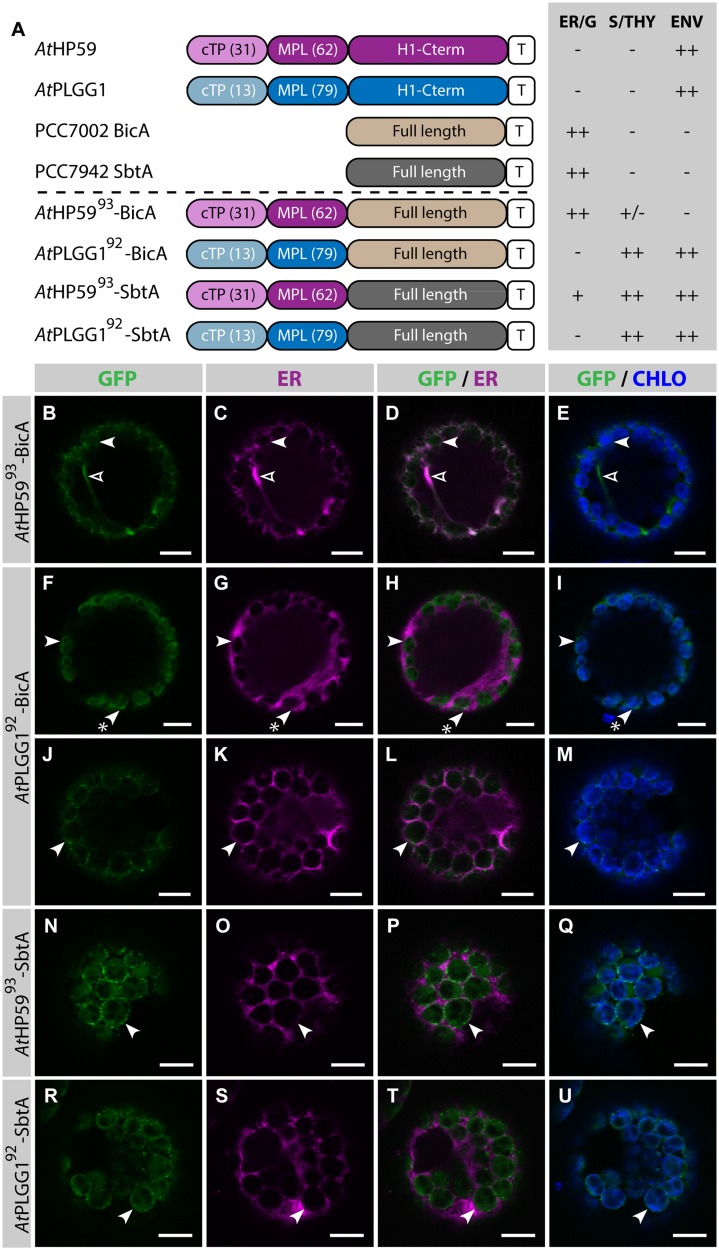
**A cTP + MPL can be sufficient to target BicA and SbtA to chloroplasts. (A)** Schematic of the chimeras used in this figure, together with a summary of their subcellular distribution as explained in **Figure 1.** ER/G, endoplasmic reticulum or Golgi apparatus; S/THY, stroma or thylakoids; ENV, chloroplast envelope; T, GFP-containing tag. Numbers in brackets indicate the number of aa making-up protein domains. **(B–U**) Single-plane confocal microscopy images of *N. benthamiana* protoplasts expressing a GFP-tagged chimera **(B,F,J,N,R)** together with an ER marker **(C,G,K,O,S)**, 2 dpi. Merges of GFP with ER **(D,H,L,P,T)** or chlorophyll signal **(E,I,M,Q,U)** are also shown. These images show that *At*HP59^93^-BicA **(B–E)** was mainly found in the ER (empty arrowheads) with traces only detected in chloroplasts (arrowheads), while *At*PLGG1^92^-BicA **(F–M)**, *At*HP59^93^-SbtA **(N–Q)** and *At*PLGG1^92^-SbtA **(R–U)** localized in chloroplasts (arrowheads). *At*PLGG1^92^-BicA, *At*HP59^93^-SbtA and *At*PLGG1^92^-SbtA were found in the chloroplast envelope as well as inside said organelles. The GFP signal in the envelope was diffused and formed stromules (starred arrowheads in **F–I**), or concentrated in foci **(J–U)**. The occurrence of both distribution patterns has been quantified in Supplementary Figure S6. Scales bars: 10 μm.

Given the results presented above, it was hypothesized that the cTP of *At*HP59 or *At*PLGG1 would be insufficient to direct BicA or SbtA to chloroplasts, but that the use of a cTP+MPL sequence might be adequate (**Figure 6**). Indeed, *At*HP59^cTP^-BicA and *At*PLGG1^cTP^-BicA localized in the ER (**Figures 6B–I**) and *At*HP59^cTP^-SbtA and *At*PLGG1^cTP^-SbtA localized in small foci suspected to be the Golgi apparatus, with traces of signal visible in the ER (**Figures 6J–Q**). We then tested the ability of the cTP+MPL of either *At*HP59 (*At*HP59^93^) or *At*PLGG1 (*At*PLGG1^92^) to redirect BicA or SbtA to chloroplasts (**Figure 7**). Traces of *At*HP59^93^-BicA were detected in chloroplasts, while most of the protein accumulated in the ER (**Figures 7B–E**). In contrast to this, *At*PLGG1^92^-BicA, *At*HP59^93^-SbtA and *At*PLGG1^92^-SbtA were mainly targeted to chloroplasts (**Figures 7F–U**). These three chimeras were found both in the envelope, were they formed stromules (examples in **Figures 7F–I**) or foci (examples in **Figures 7J–U**), and inside plastids. Envelope-localized GFP foci were observed in a subset of protoplasts expressing either of the chloroplast-targeted BicA or SbtA chimeras (Supplementary Figure S6). The significance of these foci in the envelope is unclear and might be related to protein expression level.

SbtA is an unusual transmembrane protein in that its N- and C-termini are both outside the cell in cyanobacteria (Price et al., 2011b). Here, it is unknown whether envelope-localized *At*HP59^93^-SbtA and *At*PLGG1^92^-SbtA had both N- and C-termini in the intermembrane space. To test whether the number of TMDs had an impact on the targeting of SbtA to the chloroplast, the localization of *At*HP59^145^-SbtA and *At*PLGG1^121^-SbtA, which also included the first TMD and the first intermembrane loop of each plant transporter, was assessed (Supplementary Figure S7). Both *At*HP59^145^-SbtA and *At*PLGG1^121^-SbtA were found to localize in a similar fashion to *At*HP59^93^-SbtA and *At*PLGG1^92^-SbtA, respectively, suggesting that adding an extra TMD had no effect on chloroplast targeting of SbtA chimeras.

**FIGURE 8:**
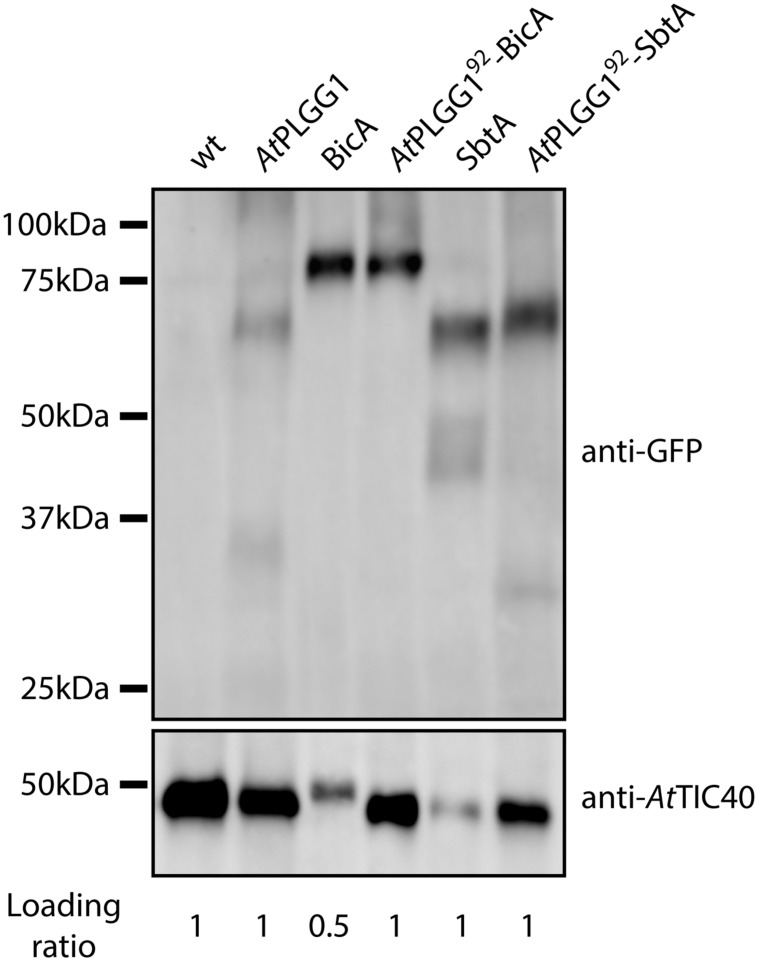
**GFP-tagged *At*PLGG1^92–^BicA and *At*PLGG1^92–^SbtA chimeras can be isolated from membranes of infiltrated *N. benthamiana* leaves.** Western-blot of membrane-enriched fractions prepared from leaves transiently expressing no GFP-tagged protein, *At*PLGG1-GFP, BicA-GFP, *At*PLGG1^92^-BicA-GFP, SbtA-GFP or *At*PLGG1^92^-SbtA-GFP, 2 dpi. The two PVDF membranes were individually blotted with an antibody against GFP to detect the chimeras and with an antibody against *At*TIC40 to confirm the presence of chloroplast envelope in the samples. Samples were loaded on a volume base, and the loading ratios are indicated under the blots. A single band was detected for each sample, confirming membrane-integration of each of the chimeras and indicating the absence of significant degradation products. Predicted size of chimeras: *At*PLGG1-GFP (∼84 kDa), BicA-GFP (∼89.6 kDa), *At*PLGG1^92^-BicA-GFP (∼99.8 kDa), SbtA-GFP (∼69.5 kDa), and *At*PLGG1^92^-SbtA-GFP (∼79.9 kDa).

**FIGURE 9:**
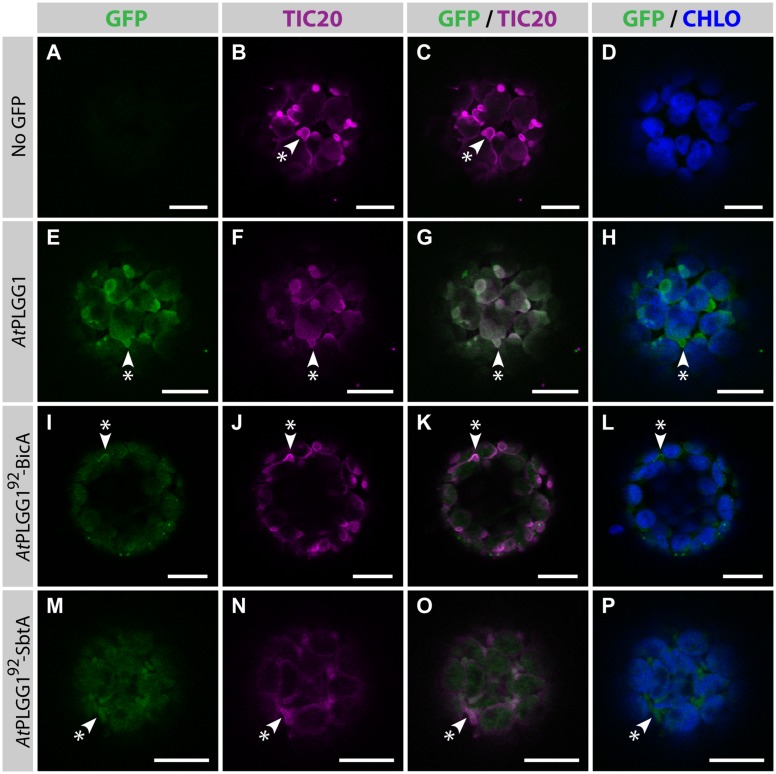
***At*PLGG1^92–^BicA and *At*PLGG1^92–^SbtA partially co-localize with the IEM protein *At*TIC20-II and form stromules in protoplasts. (A–P)** Single-plane confocal microscopy images of *N. benthamiana* protoplasts expressing a GFP-tagged chimera **(E,I,M)** or none **(A)**, together with the IEM marker *At*TIC20-II **(B,F,J,N)**, 2 dpi. Merges of GFP with *At*TIC20-II **(C,G,K,O)** or chlorophyll signal **(D,H,L,P)** are also shown. These images show that the IEM protein *At*PLGG1 **(E–H)** co-localized entirely with *At*TIC20-II including in stromules (starred arrowheads), while the envelope signal of *At*PLGG1^92^-BicA **(I–L)** and *At*PLGG1^92^-SbtA **(M–P)** co-localized with *At*TIC20-II, especially in stromules (starred arrowheads). Scales bars: 10 μm.

The detection of signal inside chloroplasts may be due to the chimeras diffusing or being translocated into thylakoid membranes, or alternatively to the presence of GFP-tagged protein degradation products in the stroma. Protein degradation was tested by western blot analysis of membrane-enriched fractions prepared from leaves transiently expressing GFP-tagged *At*PLGG1, BicA, *At*PLGG1^92^-BicA, SbtA, and *At*PLGG1^92^-SbtA (**Figure 8**). This experiment confirmed that these proteins were present in membranes and no significant degradation products were detected, suggesting that the GFP signal of *At*PLGG1, *At*PLGG1^92^-BicA, and *At*PLGG1^92^-SbtA originates from a single full-length (or near full-length) chimera (**Figure 8**). Finally, to confirm envelope localization, these three chimera were co-expressed with the IEM protein *At*TIC20-II fused to the blue fluorescent protein mTURQUOISE-II (**Figure 9**). As expected, *At*PLGG1 co-localized perfectly with *At*TIC20-II (**Figures 9E–H**). The presence of *At*PLGG1^92^-BicA and *At*PLGG1^92^-SbtA in the envelope as well as inside the chloroplast meant that these chimeras overlapped substantially, but not entirely, with *At*TIC20-II, and co-localization was visible in stromules, for example (**Figures 9I–P**).

Finally, to test whether other plant transporters could be used to target large foreign multi-pass proteins to chloroplasts, the ability of the cTP + MPL of *At*NTT1 to redirect BicA was tested (Supplementary Figure S8). *At*NTT1^115^-BicA was efficiently targeted to chloroplasts, with only traces of GFP detected in the ER (Supplementary Figures S8B–E). Similarly to *At*PLGG1^92^-BicA, *At*NTT1^115^-BicA was found inside chloroplasts and in the envelope, where it formed stromules, indicating that the cTP + MPL of either protein can be used to target BicA to the chloroplast envelope. It is therefore possible that the cTP + MPL from other large multi-pass IEM plant proteins be used to target large membrane proteins from non-chloroplastic organisms to the chloroplast envelope.

## Discussion

### First Evidence of Chloroplast Envelope Targeting of Multi-Pass Proteins from Non-Chloroplastic Organisms

To carry out a similar function in higher plants, the cyanobacterial bicarbonate transporters BicA and SbtA need to be localized in the chloroplast IEM. Prior to our study, how to target foreign nuclear-encoded membrane proteins to the chloroplast envelope was unknown. First, TPs previously known to target soluble cargos to the stroma were tested for their ability to send large membrane proteins such as BicA and SbtA to the envelope. We found that TPs from either RBCS or *At*NTT1 failed to direct BicA or SbtA to the chloroplast (**Figures 2** and **3**; Supplementary Figure S3). We then turned to plant IEM multi-pass proteins in the hope of identifying common features that would enable correct targeting of cyanobacterial transporters. Interestingly, large transporters located in the IEM possess a long N-terminal peptide (∼90–115 aa) in front of their first TMD, which we suspected may contain information sufficient to direct foreign membrane proteins to chloroplasts (see Discussion below).

We focused on two of these proteins (*At*HP59 and *At*PLGG1) and found that both their cTP and MPL were essential for their chloroplast targeting (**Figure 4**). In the N-terminus of *At*HP59, the charges are distributed asymmetrically, while this is not the case in the N-terminus of *At*PLGG1 (see Supplementary Figure S5 and discussion below). When the MPL sequence of *At*HP59 was inverted, it led to both a change in charge distribution, as well as a disruption of chloroplast-targeting (**Figures 5B–E**; Supplementary Figure S5). In the case of *At*PLGG1, inverting the MPL orientation largely maintained both charge distribution and chloroplast envelope targeting (**Figures 5F–I**; Supplementary Figure S5). Interestingly, swapping the MPL of *At*HP59 and *At*PLGG1, although affecting both N-terminus length and targeting efficiency, did not prevent chloroplast targeting, indicating that MPLs might be used in a different sequence context (see **Figures 5J–Q**; Supplementary Figure S5 and discussion below). N-terminal fragments from several large membrane IEM proteins were tested for their ability to target BicA (14 TMDs) and SbtA (10 TMDs) to chloroplasts. As expected, a cTP alone was not sufficient (**Figures 3F–I** and **6**). In contrast, the cTP + MPL of either *At*PLGG1 or *At*NTT1 was able to direct the large BicA protein to chloroplast envelopes, while the N-terminus of either *At*PLGG1 or *At*HP59 was capable of sending SbtA to chloroplast envelopes (**Figure 7**; Supplementary Figure S8). These results constitute the first instance of chloroplast envelope targeting of large non-chloroplast transmembrane proteins and provide a significant advance in chloroplast engineering *via* nuclear transformation.

Reviewing recent progress in expressing algal chloroplast proteins in plants, in light of our results, allows to discuss and finally revise the properties of the N-terminus of large plant IEM membrane proteins as well as the possible role of TMDs in chloroplast sub-compartment targeting.

### Expression of *Chlamydomonas reinhardtii* CCM Proteins in Plant Chloroplasts

In a recent study, several proteins involved in the CCM of *Chlamydomonas reinhardtii* were introduced into higher plants (Atkinson et al., 2015). Unlike cyanobacteria, *C. reinhardtii* cells harbor a chloroplast and all of its known CCM genes are encoded in the nucleus. Consequently, algal CCM proteins located inside the chloroplast or in its envelope contain chloroplast targeting signals which may enable such proteins to localize to equivalent sub-compartments in plants. In fact, Atkinson et al. (2015) showed that algal proteins located in the chloroplast envelope or around the pyrenoid in *C. reinhardtii* are targeted to chloroplasts in Tobacco. Among those is the bicarbonate transporter *Cr*Nar1.2 (*Cr*LCIA). However, two of the algal multi-pass transporters (*Cr*LCI1 and *Cr*HLA3) are located in the plasma membrane both in *C. reinhardtii* and Tobacco (Atkinson et al., 2015).

In an attempt to redirect these proteins to the chloroplast in plants, the first 60 aa of the chloroplast protein *At*ABCI13, which does not contain TMDs, were fused to *Cr*LCI1 or *Cr*HLA3. This N-terminal fragment of *At*ABCI13 contains the predicted cTP (46 aa) plus the beginning of the mature protein (14 aa). While this fragment was able to direct the small protein *Cr*LCI1 inside the chloroplast, it failed to do so with the very large protein *Cr*HLA3 (1325 aa), this chimera remaining in the plasma membrane in Tobacco (Atkinson et al., 2015). This discrepancy suggests that the TP of *At*ABCI13 is rather weak and that *Cr*HLA3 is harder to redirect than *Cr*LCI1, possibly because of its size.

In our study, we found that *At*PLGG1 could target both BicA and SbtA to the chloroplast envelope, while *At*HP59 was only able to send the smaller SbtA protein to the envelope (**Figure 7**). Notably, and unlike SbtA, BicA has a large sulfate transporter anti-sigma antagonist (STAS) domain at its C-terminus, which could render its correct targeting *in planta* harder than that of SbtA (Price and Howitt, 2011). Strikingly, in the study by Atkinson et al. (2015), although *Cr*LCI1 could be redirected to chloroplasts, it was located inside said organelles, rather than in their envelope. The authors suggested that the chimera localized in the stroma, in which case it is likely to be misfolded and non-functional given that it is a transmembrane protein. Another possibility is that the protein resides in the thylakoid membranes. In any case, this observation suggests one of the two following: the chimera either lacks IEM-retention signals or contains other motifs directing it to another chloroplast sub-compartment (see discussion below).

### Properties of the N-terminus of large IEM Transmembrane Proteins

The three cTP + MPL tested in this study, namely that of *At*HP59, *At*PLGG1 and *At*NTT1, all have a similar length (93, 92, and 115 aa, respectively). The presence of a long N-terminal fragment is a feature shared with the other 5 IEM-located membrane proteins mentioned in the results section as possessing 10 or more TMDs and a cTP. To this list was added the Na^+^/H^+^ antiporter *At*NHD1. Although there is a lack of proteomics data to confirm its presence in the IEM, it harbors a cTP, has more than 10 TMDs and a recent study used microscopy to demonstrate that it resides in the chloroplast envelope (Müller et al., 2014). Finally, *Cr*Nar1.2, the chloroplast *Chlamydomonas* bicarbonate transporter which localizes in the chloroplast envelope when expressed in higher plants, was also added to the present list as it harbors an N-terminal fragment of similar length.

**FIGURE 10:**
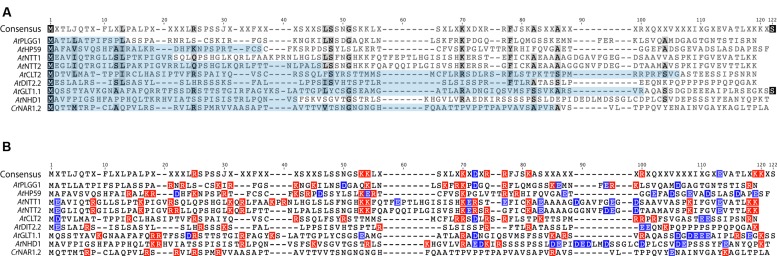
**Alignment of the cTP + MPL regions of known *A. thaliana* IEM transporters and *Cr*NAR1.2. (A)** Alignment showing poor conservation of the cTP (pale blue) and MPL regions. **(B)** same alignment showing the distribution of positive (red) and negative (blue) charges. All *A. thaliana* proteins display an asymmetric charge distribution which is not present in *Cr*NAR1.2. The end of each MPL was determined by the position of the first TMD, according to the prediction program SCAMPI-msa (see material and methods). Protein references are as follows: *At*PLGG1 (AT1G32080), *At*HP59 (AT5G59250), *At*NTT1 (AT1G80300), *At*GLT1 (AT5G16150), *At*NTT2 (AT1G15500), *At*DIT2;2 (AT5G64280), *At*CLT2 (AT4G24460), *At*NHD1 (AT3G19490) and *Cr*NAR1.2 (AAT39454).

In order to investigate the eventual presence of a conserved motif in the N-terminus of these nine proteins, their cTP + MPL fragments were aligned (**Figure 10**, *At*PHT2;1 was left out of this alignment as its N-terminus is unusually rich in histidines). Although they share a similar length, the cTP + MPL sequence of these nine proteins was poorly conserved (**Figure 10A**). This is in line with previous knowledge that cTPs vary in length and that their aa sequence is poorly conserved (Li and Chiu, 2010). Detailed studies of the TP from RBCS and chlorophyll a/b- binding protein (CAB) also revealed that different fragments of the TP are involved in various steps of chloroplast import; consequently, successful import of soluble proteins relies on the length and general aa context of the TP sequence, rather than on the presence of a single motif (Lee et al., 2002, 2006, 2015). Albeit poorly conserved, the cTP+MPL of the 8 plant proteins appeared asymmetrically charged; their N-terminus being dominated by positive charges, while their C-terminus harbored more negative charges (**Figure 10B**). Although the functionality of this charge asymmetry is unclear, we have observed that its disruption in *At*HP59 resulted in a loss of chloroplast targeting (**Figure 5**; Supplementary Figure S5). This raises the interesting possibility that charge distribution might be involved in the targeting process of large IEM transmembrane proteins in plants. Notably, the cTP + MPL of *Cr*NAR1.2 was not charged, suggesting that in *Chlamydomonas* the mechanism regulating import of equivalent proteins might operate differently, but still enables correct targeting in higher plants (**Figure 10B**). Future work will be necessary to determine the relative importance of charge distribution in the chloroplast import process of IEM proteins.

### Envelope or Thylakoid Targeting: Putative Role of TMDs in BicA and SbtA

In our study, when redirected to the chloroplast, BicA and SbtA localized in the chloroplast envelope as well as inside chloroplasts, which we speculate to result from the presence of part of the protein pool in thylakoid membranes. This might be due to information contained within BicA and SbtA and an interesting possibility is that TMDs are involved in this sub-compartment targeting. In an elegant study, Froehlich and Keegstra swapped the TMD of the single-pass IEM protein “accumulation and replication of chloroplasts 6” (ARC6) with that of the single-pass thylakoid membrane proteins “state transition protein kinase 8” (STN8) or “plastidic type I signal peptidase 1” (PLSP1) (Froehlich and Keegstra, 2011). Interestingly, this was sufficient to send the IEM protein to the thylakoid membrane and *vice versa*, indicating that once the proteins are in the chloroplast, the TMDs are sufficient to determine in which membrane the proteins localize (Froehlich and Keegstra, 2011). The situation is more complicated in the case of chloroplast-targeted BicA and SbtA for mainly two reasons: (1) they have 14 and 10 TMDs, respectively, and (2) their sequence evolved to be localized in the plasma membrane in cyanobacteria. In plant chloroplasts, the IEM is connected to thylakoid membranes in many points such that these two membrane compartments are in fact a continuum (Rosado-alberio et al., 1968). It is therefore tempting to speculate that the N-terminal 92 aa of a plant transporter such as *At*PLGG1 are sufficient for BicA and SbtA to reach the envelope, and that once in the inner chloroplast membrane continuum, the sequence of the cyanobacterial transporter determines whether it localizes in the IEM or the thylakoid membrane.

### The cTP is a Fixed Sequence While the TP Depends on its Cargo

The length of the cTP is determined by the SPP cleavage site (Richter et al., 2005). In this sense, it is considered a fixed sequence. Contrastingly, we observed that (1) membrane proteins require different targeting signals than soluble proteins and that (2) the cTP + MPL sequence of *At*PLGG1 worked for both BicA and SbtA while that of *At*HP59 only worked for SbtA. Our results suggest that the sequence sufficient to target a protein to the chloroplast (TP) depends on its cargo. In light of our results, TPs should be regarded as variable sequences, where length and structure need to be adapted to their cargos, and the chloroplast targeting efficiency of foreign nuclear-encoded proteins needs to be individually evaluated.

## Author Contributions

VR, MB, and GP designed the research; VR performed the research; VR, MB, and GP analyzed the data; VR drafted the manuscript, VR, MB, and GP contributed to the final manuscript.

## Conflict of Interest Statement

The authors declare that the research was conducted in the absence of any commercial or financial relationships that could be construed as a potential conflict of interest.

## Footnotes

1 www.cbs.dtu.dk/services/TargetP/

2 www.cbs.dtu.dk/services/ChloroP/

3 topcons.cbr.su.se

4 www.grenoble.prabi.fr/at_chloro

5 abrc.osu.edu

## Abbreviations

CCM: CO_2_-concentrating mechanism
IEM: inner envelope membrane
MPL: membrane protein leader
OEM: outer envelope membrane
TIC: translocon at the inner envelope membrane of chloroplasts
TMD: transmembrane domain
TOC: translocon at the outer envelope membrane of chloroplasts
TP: transit peptide

## References

[B1] Atkinson N. Feike D. Mackinder L. C. M. Meyer M. T. Griffiths H. Jonikas M. C. (2015). Introducing an algal carbon-concentrating mechanism into higher plants: location and incorporation of key components. *Plant Biotechnol. J.* 10.1111/pbi.12497 [Epub ahead of print]. PMC5102585 26538195

[B2] Bernsel A. Viklund H. Falk J. Lindahl E. von Heijne G. Elofsson A. (2008). Prediction of membrane-protein topology from first principles. *Proc. Natl. Acad. Sci. U.S.A.* 105 7177–7181. 10.1073/pnas.0711151105 18477697 PMC2438223

[B3] Bionda T. Tillmann B. Simm S. Beilstein K. Ruprecht M. Schleiff E. (2010). Chloroplast import signals: the length requirement for translocation in vitro and in vivo. *J. Mol. Biol.* 402 510–523. 10.1016/j.jmb.2010.07.052 20688079

[B4] Breuers F. K. H. Bräutigam A. Geimer S. Welzel U. Y. Stefano G. Renna L. (2012). Dynamic remodeling of the plastid envelope membranes - a tool for chloroplast envelope in vivo localizations. *Front. Plant Sci.* 3:7 10.3389/fpls.2012.00007 PMC3355811 22645566

[B5] Cao M.-J. Wang Z. Wirtz M. Hell R. Oliver D. J. Xiang C.-B. (2013). SULTR3;1 is a chloroplast-localized sulfate transporter in *Arabidopsis thaliana*. *Plant J.* 73 607–616. 10.1111/tpj.12059 23095126

[B6] Chua N.-H. Schmidt G. W. (1978). Post-translational transport into intact chloroplasts of a precursor to the small subunit of ribulose-1,5-bisphosphate carboxylase. *Proc. Natl. Acad. Sci. U.S.A.* 75 6110–6114. 10.1073/pnas.75.12.6110 16592597 PMC393128

[B7] Comai L. Larson-Kelly N. Kiser J. Mau C. J. Pokalsky A. R. Shewmaker C. K. (1988). Chloroplast transport of a ribulose bisphosphate carboxylase small subunit-5-enolpyruvyl 3-phosphoshikimate synthase chimeric protein requires part of the mature small subunit in addition to the transit peptide. *J. Biol. Chem.* 263 15104–15109. 3049600

[B8] Curtis M. D. Grossniklaus U. (2003). A gateway cloning vector set for high-throughput functional analysis of genes in planta. *Plant Physiol.* 133 462–469. 10.1104/pp.103.027979 14555774 PMC523872

[B9] Dobberstein B. Blobel G. Chua N. H. (1977). In vitro synthesis and processing of a putative precursor for the small subunit of ribulose-1,5-bisphosphate carboxylase of *Chlamydomonas reinhardtii*. *Proc. Natl. Acad. Sci. U.S.A.* 74 1082–1085. 10.1073/pnas.74.3.1082 265554 PMC430598

[B10] Du J. Förster B. Rourke L. Howitt S. M. Price G. D. (2014). Characterisation of cyanobacterial bicarbonate transporters in *E. coli* shows that SbtA homologs are functional in this heterologous expression system. *PLoS ONE* 9:e115905 10.1371/journal.pone.0115905 PMC4275256 25536191

[B11] Emanuelsson O. Brunak S. von Heijne G. Nielsen H. (2007). Locating proteins in the cell using TargetP, SignalP and related tools. *Nat. Protoc.* 2 953–971. 10.1038/nprot.2007.131 17446895

[B12] Emanuelsson O. Nielsen H. von Heijne G. (1999). ChloroP, a neural network-based method for predicting chloroplast transit peptides and their cleavage sites. *Protein Sci.* 8 978–984. 10.1110/ps.8.5.978 10338008 PMC2144330

[B13] Ferro M. Brugière S. Salvi D. Seigneurin-Berny D. Court M. Moyet L. (2010). AT_CHLORO, a comprehensive chloroplast proteome database with subplastidial localization and curated information on envelope proteins. *Mol. Cell. Proteomics* 9 1063–1084. 10.1074/mcp.M900325-MCP200 20061580 PMC2877971

[B14] Froehlich J. E. Keegstra K. (2011). The role of the transmembrane domain in determining the targeting of membrane proteins to either the inner envelope or thylakoid membrane. *Plant J.* 68 844–856. 10.1111/j.1365-313X.2011.04735.x 21838779

[B15] Goedhart J. von Stetten D. Noirclerc-Savoye M. Lelimousin M. Joosen L. Hink M. A. (2012). Structure-guided evolution of cyan fluorescent proteins towards a quantum yield of 93%. *Nat. Commun.* 3:751 10.1038/ncomms1738 PMC3316892 22434194

[B16] Hanson M. R. Gray B. N. Ahner B. A. (2013). Chloroplast transformation for engineering of photosynthesis. *J. Exp. Bot.* 64 731–742. 10.1093/jxb/ers325 23162121

[B17] Haseloff J. (1999). GFP variants for multispectral imaging of living cells. *Methods Cell Biol.* 58 139–151. 10.1016/S0091-679X(08)61953-6 9891379

[B18] Highfield P. E. Ellis R. J. (1978). Synthesis and transport of the small subunit of chloroplast ribulose bisphosphate carboxylase. *Nature* 271 420–424. 10.1038/271420a0

[B19] Koncz C. Schell J. (1986). The promoter of TL-DNA gene 5 controls the tissue-specific expression of chimaeric genes carried by a novel type of *Agrobacterium* binary vector. *Mol. Gen. Genet.* 204 383–396. 10.1007/BF00331014

[B20] Lee D. W. Lee S. Lee G. Lee K. H. Kim S. Cheong G. (2006). Functional characterization of sequence motifs in the transit peptide of *Arabidopsis* small subunit. *Plant Physiol.* 140 466–483. 10.1104/pp.105.074575.Cline 16384899 PMC1361317

[B21] Lee D. W. Woo S. Geem K. R. Hwang I. (2015). Sequence motifs in transit peptides act as independent functional units and can be transferred to new sequence contexts. *Plant Physiol.* 169 471–484. 10.1104/pp.15.00842 26149569 PMC4577419

[B22] Lee K. H. Kim D. H. Lee S. W. Kim Z. H. Hwang I. (2002). In vivo import experiments in protoplasts reveal the importance of the overall context but not specific amino acid residues of the transit peptide during import into chloroplasts. *Mol. Cells* 14 388–397. 12521302

[B23] Li H. Chiu C.-C. (2010). Protein transport into chloroplasts. *Annu. Rev. Plant Biol.* 61 157–180. 10.1146/annurev-arplant-042809-112222 20192748

[B24] Li M. Schnell D. J. (2006). Reconstitution of protein targeting to the inner envelope membrane of chloroplasts. *J. Cell Biol.* 175 249–259. 10.1083/jcb.200605162 17060496 PMC2064566

[B25] Long S. P. Marshall-Colon A. Zhu X.-G. (2015). Meeting the global food demand of the future by engineering crop photosynthesis and yield potential. *Cell* 161 56–66. 10.1016/j.cell.2015.03.019 25815985

[B26] Ludwig L. J. Canvin D. T. (1971). The rate of photorespiration during photosynthesis and the relationship of the substrate of light respiration to the products of photosynthesis in sunflower leaves. *Plant Physiol.* 48 712–719. 10.1104/pp.48.6.712 16657866 PMC396934

[B27] McGrath J. M. Long S. P. (2014). Can the cyanobacterial carbon-concentrating mechanism increase photosynthesis in crop species? A theoretical analysis. *Plant Physiol.* 164 2247–2261. 10.1104/pp.113.232611 24550242 PMC3982776

[B28] Mittler R. Blumwald E. (2010). Genetic engineering for modern agriculture: challenges and perspectives. *Annu. Rev. Plant Biol.* 61 443–462. 10.1146/annurev-arplant-042809-112116 20192746

[B29] Müller M. Kunz H. H. Schroeder J. I. Kemp G. Young H. S. Ekkehard Neuhaus H. (2014). Decreased capacity for sodium export out of *Arabidopsis* chloroplasts impairs salt tolerance, photosynthesis and plant performance. *Plant J.* 78 646–658. 10.1111/tpj.12501 24617758

[B30] Nelson B. K. Cai X. Nebenführ A. (2007). A multicolored set of in vivo organelle markers for co-localization studies in *Arabidopsis* and other plants. *Plant J.* 51 1126–1136. 10.1111/j.1365-313X.2007.03212.x 17666025

[B31] Neuhaus H. E. Thom E. Möhlmann T. Steup M. Kampfenkel K. (1997). Characterization of a novel eukaryotic ATP/ADP translocator located in the plastid envelope of *Arabidopsis thaliana* L. *Plant J.* 11 73–82. 10.1046/j.1365-313X.1997.11010073.x 9025303

[B32] Omata T. Price G. D. Badger M. R. Okamura M. Gohta S. Ogawa T. (1999). Identification of an ATP-binding cassette transporter involved in bicarbonate uptake in the cyanobacterium *Synechococcus* sp. strain PCC 7942. *Proc. Natl. Acad. Sci. U.S.A.* 96 13571–13576. 10.1073/pnas.96.23.13571 10557362 PMC23989

[B33] Ort D. R. Merchant S. S. Alric J. Barkan A. Blankenship R. E. Bock R. (2015). Redesigning photosynthesis to sustainably meet global food and bioenergy demand. *Proc. Natl. Acad. Sci. U.S.A.* 112 8529–8536. 10.1073/pnas.1424031112 26124102 PMC4507207

[B34] Pengelly J. J. L. Forster B. von Caemmerer S. Badger M. R. Price G. D. Whitney S. M. (2014). Transplastomic integration of a cyanobacterial bicarbonate transporter into tobacco chloroplasts. *J. Exp. Bot.* 65 3071–3080. 10.1093/jxb/eru156 24965541 PMC4071830

[B35] Pick T. R. Bräutigam A. Schulz M. A. Obata T. Fernie A. R. Weber A. P. M. (2013). PLGG1, a plastidic glycolate glycerate transporter, is required for photorespiration and defines a unique class of metabolite transporters. *Proc. Natl. Acad. Sci. U.S.A.* 110 3185–3190. 10.1073/pnas.1215142110 23382251 PMC3581909

[B36] Price G. D. (2011). Inorganic carbon transporters of the cyanobacterial CO_2_ concentrating mechanism. *Photosynth. Res.* 109 47–57. 10.1007/s11120-010-9608-y 21359551

[B37] Price G. D. Badger M. R. von Caemmerer S. (2011a). The prospect of using cyanobacterial bicarbonate transporters to improve leaf photosynthesis in C3 crop plants. *Plant Physiol.* 155 20–26. 10.1104/pp.110.164681 20923885 PMC3075752

[B38] Price G. D. Shelden M. C. Howitt S. M. (2011b). Membrane topology of the cyanobacterial bicarbonate transporter, SbtA, and identification of potential regulatory loops. *Mol. Membr. Biol.* 28 265–275. 10.3109/09687688.2011.593049 21688970

[B39] Price G. D. Badger M. R. Woodger F. J. Long B. M. (2008). Advances in understanding the cyanobacterial CO_2_-concentrating-mechanism (CCM): functional components, Ci transporters, diversity, genetic regulation and prospects for engineering into plants. *J. Exp. Bot.* 59 1441–1461. 10.1093/jxb/erm112 17578868

[B40] Price G. D. Howitt S. M. (2011). The cyanobacterial bicarbonate transporter BicA: its physiological role and the implications of structural similarities with human SLC26 transporters. *Biochem. Cell Biol.* 89 178–188. 10.1139/o10-136 21455269

[B41] Price G. D. Howitt S. M. (2014). Topology mapping to characterize cyanobacterial bicarbonate transporters: BicA (SulP/SLC26 family) and SbtA. *Mol. Membr. Biol.* 31 177–182. 10.3109/09687688.2014.953222 25222859

[B42] Price G. D. Pengelly J. J. L. Forster B. Du J. Whitney S. M. von Caemmerer S. (2013). The cyanobacterial CCM as a source of genes for improving photosynthetic CO_2_ fixation in crop species. *J. Exp. Bot.* 64 753–768. 10.1093/jxb/ers257 23028015

[B43] Price G. D. Woodger F. J. Badger M. R. Howitt S. M. Tucker L. (2004). Identification of a SulP-type bicarbonate transporter in marine cyanobacteria. *Proc. Natl. Acad. Sci. U.S.A.* 101 18228–18233. 10.1073/pnas.0405211101 15596724 PMC539743

[B44] Rae B. D. Long B. M. Badger M. R. Price G. D. (2013a). Functions, compositions, and evolutions of the two types of carboxysomes?: polyhedral microcompartments that facilitate CO_2_ fixation in cyanobacteria and some *Proteobacteria*. *Microbiol. Mol. Biol. Rev.* 77 1–25. 10.1128/MMBR.00061-12 24006469 PMC3811607

[B45] Rae B. D. Long B. M. Whitehead L. F. Förster B. Badger M. R. Price G. D. (2013b). Cyanobacterial carboxysomes: microcompartments that facilitate CO_2_ fixation. *J. Mol. Microbiol. Biotechnol.* 23 300–307. 10.1159/000351342 23920493

[B46] Richter S. Zhong R. Lamppa G. (2005). Function of the stromal processing peptidase in the chloroplast import pathway. *Physiol. Plant.* 123 362–368. 10.1111/j.1399-3054.2005.00476.x

[B47] Rosado-alberio J. Weier T. E. Stocking C. R. (1968). Continuity of the chloroplast membrane systems in *Zea mays* L. *Plant Physiol.* 43 1325–1331. 10.1104/pp.43.9.1325 16656915 PMC1087017

[B48] Runquist D. Hahn-Hägerdal B. Rådström P. (2010). Comparison of heterologous xylose transporters in recombinant *Saccharomyces cerevisiae*. *Biotechnol. Biofuels* 3:5 10.1186/1754-6834-3-5 PMC2851583 20236521

[B49] Shelden M. C. Howitt S. M. Price G. D. (2010). Membrane topology of the cyanobacterial bicarbonate transporter, BicA, a member of the SulP (SLC26A) family. *Mol. Membr. Biol.* 27 12–23. 10.3109/09687680903400120 19951076

[B50] Shibata M. Katoh H. Sonoda M. Ohkawa H. Shimoyama M. Fukuzawa H. (2002). Genes essential to sodium-dependent bicarbonate transport in cyanobacteria: function and phylogenetic analysis. *J. Biol. Chem.* 277 18658–18664. 10.1074/jbc.M112468200 11904298

[B51] Tripp J. Inoue K. Keegstra K. Froehlich J. E. (2007). A novel serine/proline-rich domain in combination with a transmembrane domain is required for the insertion of AtTic40 into the inner envelope membrane of chloroplasts. *Plant J.* 52 824–838. 10.1111/j.1365-313X.2007.03279.x 17883373

[B52] Tsirigos K. D. Peters C. Shu N. Kall L. Elofsson A. (2015). The TOPCONS web server for consensus prediction of membrane protein topology and signal peptides. *Nucleic Acids Res.* 37 W465–W468. 10.1093/nar/gkv485 PMC4489233 25969446

[B53] Viana A. A. B. Li M. Schnell D. J. (2010). Determinants for stop-transfer and post-import pathways for protein targeting to the chloroplast inner envelope membrane. *J. Biol. Chem.* 285 12948–12960. 10.1074/jbc.M110.109744 20194502 PMC2857071

[B54] Whitney S. M. Houtz R. L. Alonso H. (2011). Advancing our understanding and capacity to engineer nature’s CO_2_-sequestering enzyme, Rubisco. *Plant Physiol.* 155 27–35. 10.1104/pp.110.164814 20974895 PMC3075749

[B55] Zhu X.-G. Long S. P. Ort D. R. (2010). Improving photosynthetic efficiency for greater yield. *Annu. Rev. Plant Biol.* 61 235–261. 10.1146/annurev-arplant-042809-112206 20192734

