# Supplementary material for: Redirecting the Cyanobacterial Bicarbonate Transporters BicA and SbtA to the Chloroplast Envelope: Soluble and Membrane Cargos Need Different Chloroplast Targeting Signals in Plants
Source: Front Plant Sci. 2016 Feb 29;7:185. doi: 10.3389/fpls.2016.00185 (PMC4770052; doi:10.3389/fpls.2016.00185)
Supplement: Supplementary file 1 [file Image_1.pdf]

FIGURE S1

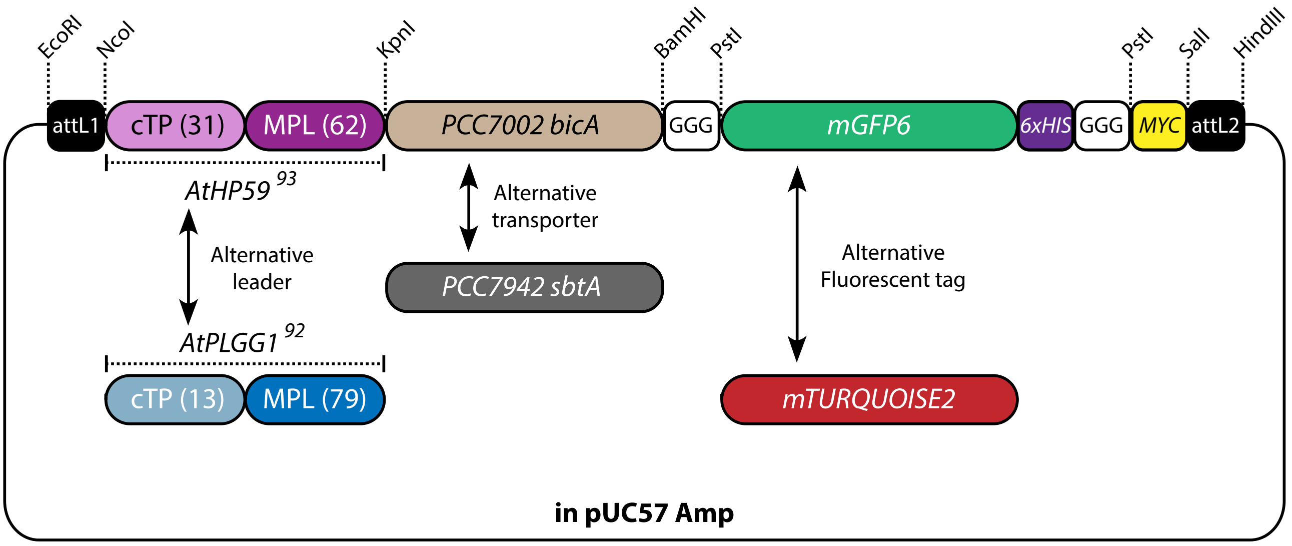

Figure S1. Diagram of At*HP59*^{93}*-BicA-mGFP6-6xHIS-MYC* in pUC57 Amp in which, the leader, the cargo, and the tag can easily be exchanged.

FIGURE S2

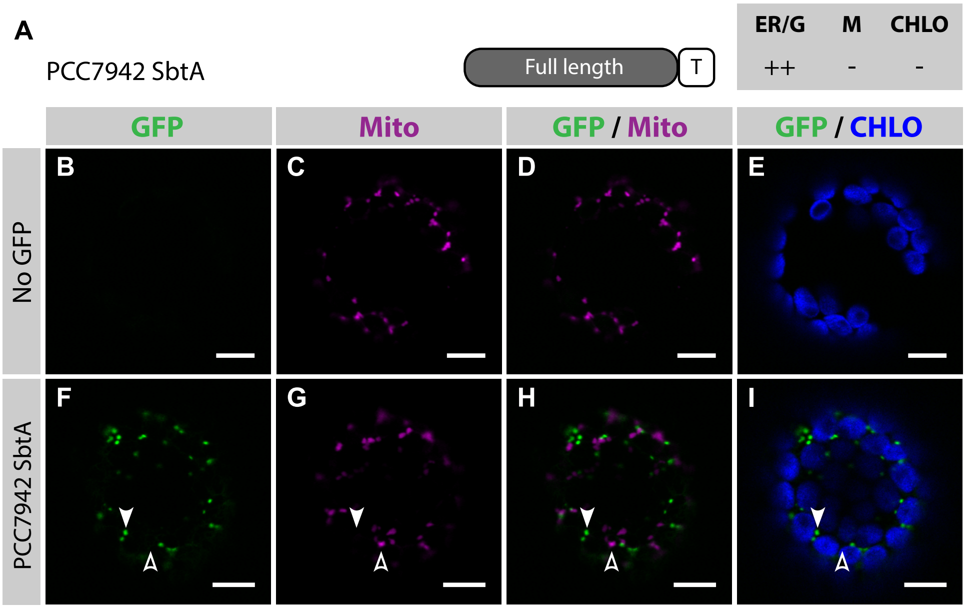

Figure S2. Nuclear-encoded SbtA does not localize in mitochondria in *N. benthamiana*.

(A) Schematic of PCC7942 SbtA, and summary of its subcellular distribution as explained in Figure 1. ER/G: endoplasmic reticulum or golgi apparatus; M: mitochondria; CHLO: chloroplast; T: GFP-containing tag. **(B-I)** Single-plane confocal microscopy images of *N. benthamiana* protoplasts expressing GFP-tagged PCC7942 SbtA (F) or not (B) together with a mitochondrial marker (C and G), 2dpi. Merges of GFP with mitochondrial (D and H) or chlorophyll signal (E, I) are also shown. These images show that PCC7942 SbtA (arrowheads in F-I) did not co-localize with the mitochondrial marker (empty arrowheads in F-I). Scales bars: 10 µm.

FIGURE S3

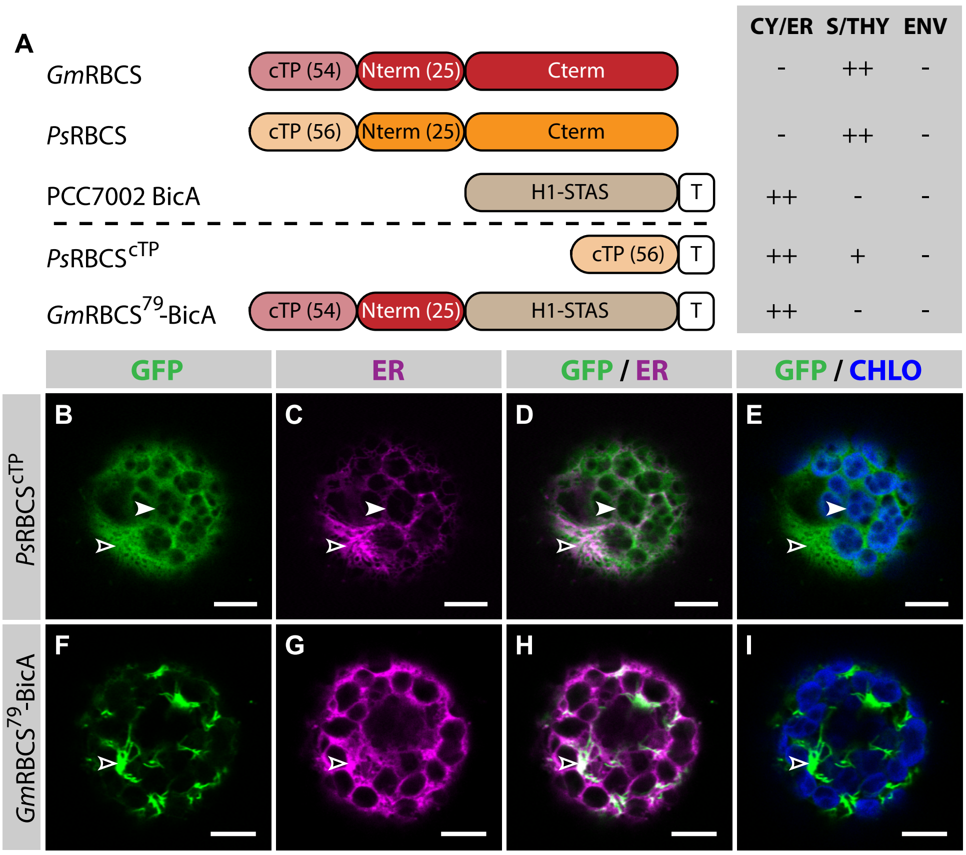

Figure S3. *Ps*RBCS^{cTP} only achieves partial chloroplastic translocation of GFP, while *Gm*RBCS^{79}-BicA cannot reach the chloroplast.

(A) Schematic of the RBCS/BicA chimeras used in this figure together with a summary of their subcellular distribution as explained in Figure 1. CY/ER: cytosol or endoplasmic reticulum; S/THY: stroma or thylakoids; ENV: chloroplast envelope; T: GFP-containing tag. Numbers in brackets indicate the number of aa making-up protein domains. The subcellular localization of *Gm*RBCS and *Ps*RBCS were inferred from the literature. **(B-I)** Single-plane confocal microscopy images of *N. benthamiana* protoplasts expressing a GFP-tagged chimera (B and F) together with an ER marker (C and G), 2dpi. Merges of GFP with ER (D, H) or chlorophyll signal (E, I) are also shown. These images show that *Ps*RBCS^{cTP} localized primarily in the cytosol (empty arrowheads in B-E) and secondarily in chloroplasts (arrowheads in B-E) while *Gm*RBCS^{79}-BicA accumulated outside chloroplasts (empty arrowheads in F-I). Scales bars: 10 µm.

FIGURE S4

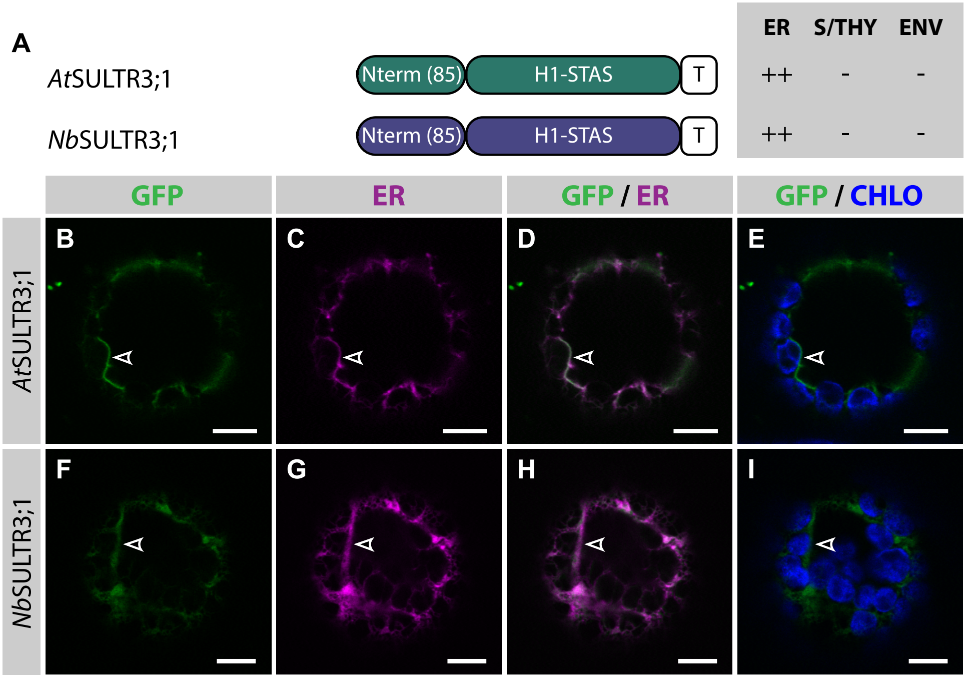

Figure S4. *At*SULTR3;1 and *Nb*SULTR3;1 do not localize in chloroplasts in *N. benthamiana* protoplasts.

(A) Schematic of *At*SULTR3;1 and *Nb*SULTR3;1 together with a summary of their subcellular distribution as explained in Figure 1. ER: endoplasmic reticulum; S/THY: stroma or thylakoids; ENV: chloroplast envelope; T: GFP-containing tag. Numbers in brackets indicate the number of aa making-up protein domains. **(B-I)** Single-plane confocal microscopy images of *N. benthamiana* protoplasts expressing a GFP-tagged chimera (B and F) together with an ER marker (C and G), 2 dpi. Merges of GFP with ER (D and H) or chlorophyll signal (E and I) are also shown. These images show that *At*SULTR3;1 (B-E) and *Nb*SULTR3;1 (F-I) localized in the ER (empty arrowheads). Scales bars: 10 µm.

FIGURE S5

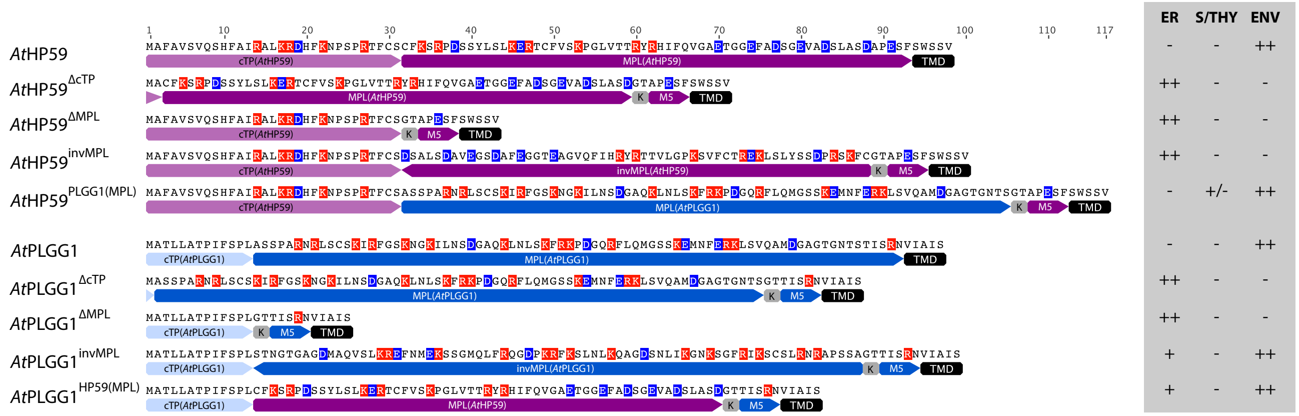

Figure S5. Charge distribution in the N-terminus of *At*HP59 and *At*PLGG1 chimeras presented in Figure 4 and Figure 5.

Positively- and negatively-charged amino acids are highlighted in red and blue, respectively. TMD: beginning of the first transmembrane domain; M5: last 5 amino acids of the MPL; K: KpnI restriction site. For sake of clarity the subcellular distribution of the different chimeras presented in Figure 4 and Figure 5 has been added on the right hand side of the figure. Note that in *At*HP59^{invMPL}, which was not targeted to the chloroplast, the positive stretch of positive charges present in *At*HP59 was interrupted by a series of negative charges. This was not the case in *At*PLGG1^{invMPL}, where positive and negative charges are more evenly distributed.

FIGURE S6

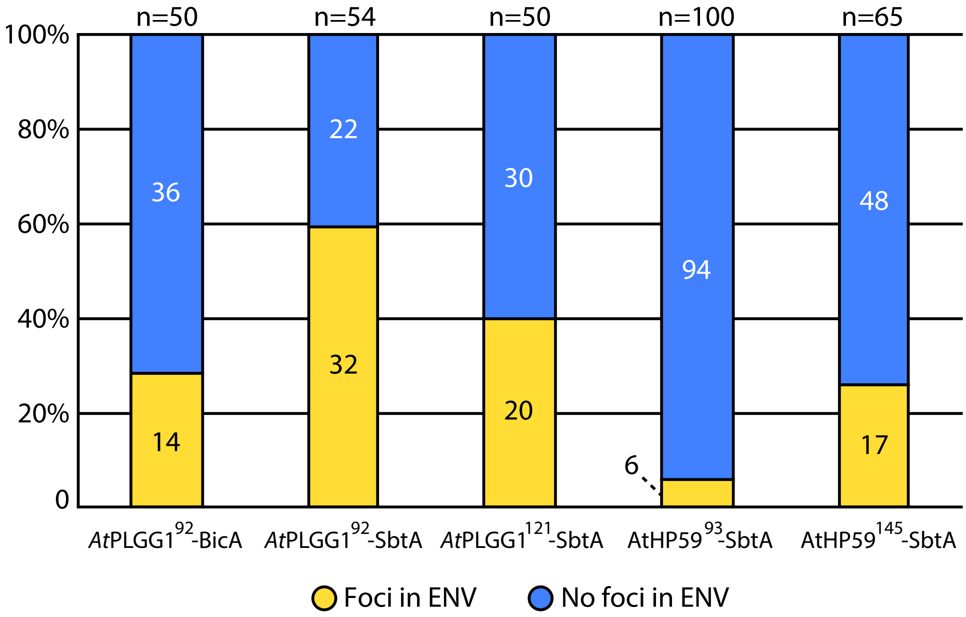

Figure S6. Quantification of the presence/absence of foci in the envelope of chloroplast-localized SbtA and BicA chimeras.

The percentage of protoplasts in which the chloroplast envelope GFP signal was localized in foci (yellow bars) or not (blue bars) has been calculated for *At*PLGG1^{92}-BicA, *At*PLGG1^{92}-SbtA, *At*PLGG1^{121}-SbtA, *At*HP59^{93}-SbtA and *At*HP59^{145}-SbtA from 50 to 100 protoplasts. The total number of protoplasts counted for each construct is indicated above each bar, and the number of protoplasts making-up each fraction in indicated inside each bar. All measurements were done 2 dpi.

FIGURE S7

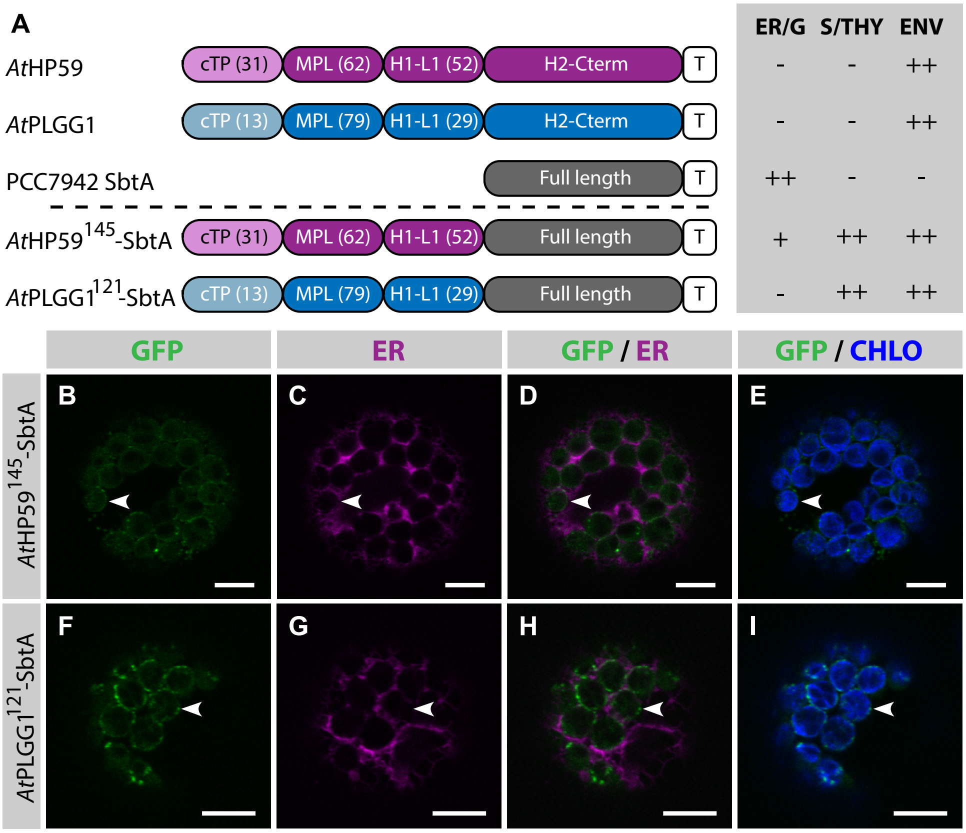

Figure S7. *At*HP59^{145}-SbtA and *At*PLGG1^{121}-SbtA are targeted to chloroplasts.
(A) Schematic of *At*HP59^{145}-SbtA and *At*PLGG1^{121}-SbtA together with a summary of their subcellular distribution as explained in figure 1. ER: endoplasmic reticulum; S/THY: stroma or thylakoids; ENV: chloroplast envelope; T: GFP-containing tag. Numbers in brackets indicate the number of aa making-up protein domains. **(B-I)** Single-plane confocal microscopy images of *N. benthamiana* protoplasts expressing a GFP-tagged chimera (B and F) together with an ER marker (C and G), 2 dpi. Merges of GFP with ER (D and H) or chlorophyll signal (E and I) are also shown. These images show that both *At*HP59^{145}-SbtA (B-E) and *At*PLGG1^{121}-SbtA (F-I) localized in the chloroplast envelope (arrowheads) as well as inside chloroplasts. Scales bars: 10 µm.

FIGURE S8

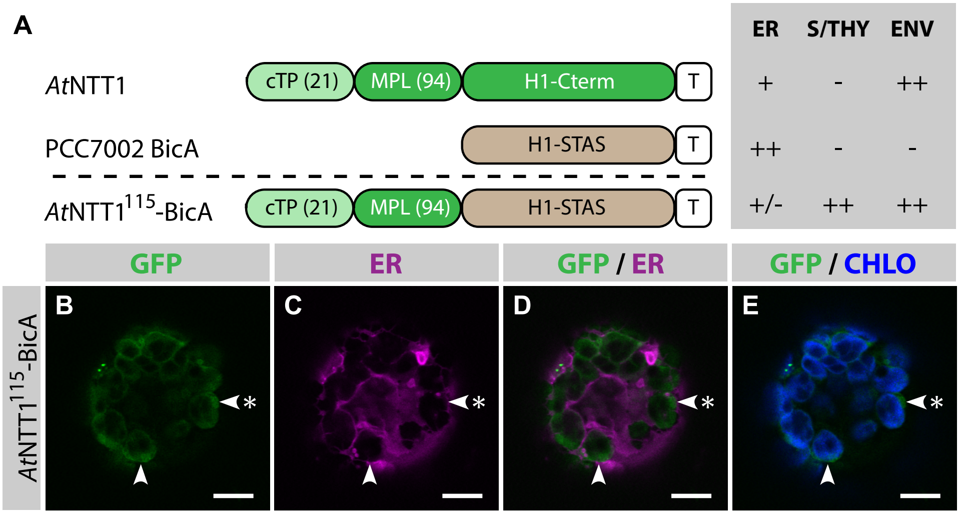

Figure S8. The cTP+MPL of *At*NTT1 is able to target BicA to chloroplasts where it forms stromules.

(A) Schematic of *At*NTT1^{115}-BicA together with a summary of its subcellular distribution as explained in figure 1. ER: endoplasmic reticulum; S/THY: stroma or thylakoids; ENV: chloroplast envelope; T: GFP-containing tag. Numbers in brackets indicate the number of aa making-up protein domains. **(B-E)** Single-plane confocal microscopy images of *N. benthamiana* protoplasts expressing a GFP-tagged *At*NTT1^{115}-BicA (B) together with an ER marker (C), 2 dpi. Merges of GFP with ER (D) or chlorophyll signal (E) are also shown. These images show that both *At*NTT1^{115}-BicA was targeted to chloroplasts (arrowheads), including their envelope were it formed stromules (starred arrowheads). Scale bars: 10 µm.

	Construct name	Plasmid of origin	PCR Primers	Digested with	Resulting insert	Into	Name of entry vector	Destination vector
Cloning method 1	PsRBCS^{cTP}	---	---	---	---	---	PsRBCS^{cTP}-mGFP6-6xHIS-MYC_pUC57 Amp	PsRBCS^{cTP}-mGFP6-6xHIS-MYC_pMDC32
	GmRBCS^{cTP}	---	---	---	---	---	GmRBCS^{cTP}-mGFP6-6xHIS-MYC_pUC57 Amp	GmRBCS^{cTP}-mGFP6-6xHIS-MYC_pMDC32
	GmPsRBCS^{79}	---	---	---	---	---	GmPsRBCS^{79}-mGFP6-6xHIS-MYC_pUC57 Amp	GmPsRBCS^{79}-mGFP6-6xHIS-MYC_pMDC32
	GmPsRBCS^{79}-BicA	---	---	---	---	---	GmPsRBCS^{79}-BicA-mGFP6-6xHIS-MYC_pUC57 Amp	GmPsRBCS^{79}-BicA-mGFP6-6xHIS-MYC_pMDC32
	AtHP59^{93}-BicA	---	---	---	---	---	AtHP59^{93}-BicA-mGFP6-6xHIS-MYC_pUC57 Amp	AtHP59^{93}-BicA-mGFP6-6xHIS-MYC_pMDC32
	AtTIC20-II	---	---	---	---	---	AtTIC20-II-mTURQUOISE2-6xHIS-MYC_pUC57 Amp	AtTIC20-II-mTURQUOISE2-6xHIS-MYC_pMDC32
Cloning method 2	PCC7002 BicA	PCC7002 BicA-mGFP6-6xHIS-MYC_pUC57 Amp	52/27	---	PCC7002 BicA-mGFP6-6xHIS-MYC	pDNOR207	PCC7002 BicA-mGFP6-6xHIS-MYC_pDNOR207	PCC7002 BicA-mGFP6-6xHIS-MYC_pMDC32
	AtPLGG1	AtPLGG1-mGFP6-6xHIS-MYC_pUC57 Amp	28/27	---	AtPLGG1-mGFP6-6xHIS-MYC	pDNOR207	AtPLGG1-mGFP6-6xHIS-MYC_pDNOR207	AtPLGG1-mGFP6-6xHIS-MYC_pMDC32
	AtSULTR3;1	AtSULTR3;1-mGFP6-6xHIS-MYC_pUC57 Kan	30/27	---	AtSULTR3;1-mGFP6-6xHIS-MYC	pDNOR207	AtSULTR3;1-mGFP6-6xHIS-MYC_pDNOR207	AtSULTR3;1-mGFP6-6xHIS-MYC_pMDC32
	PCC7942 SbtA	PCC7942 SbtA-mGFP6-6xHIS-MYC_pUC57 Kan	68/27	---	PCC7942 SbtA-mGFP6-6xHIS-MYC	pDNOR221	PCC7942 SbtA-mGFP6-6xHIS-MYC_pDNOR221	PCC7942 SbtA-mGFP6-6xHIS-MYC_pMDC32
	AtNTT1	AtNTT1-mGFP6-6xHIS-MYC_pUC57 Amp	98/27	---	AtNTT1-mGFP6-6xHIS-MYC	pDNOR221	AtNTT1-mGFP6-6xHIS-MYC_pDNOR221	AtNTT1-mGFP6-6xHIS-MYC_pMDC32
	AtHP59	AtHP59-mGFP6-6xHIS-MYC_pUC57 Amp	26/27	---	AtHP59-mGFP6-6xHIS-MYC	pDNOR221	AtHP59-mGFP6-6xHIS-MYC_pDNOR221	AtHP59-mGFP6-6xHIS-MYC_pMDC32
	NbSULTR3;1	NbSULTR3;1-mGFP6-6xHIS-MYC_pUC57 Amp	31/27	---	NbSultr3;1-mGFP6-6xHIS-MYC	pDNOR221	NbSULTR3;1-mGFP6-6xHIS-MYC_pDNOR221	NbSULTR3;1-mGFP6-6xHIS-MYC_pMDC32
Cloning method 3	GmRBCS^{79}-BicA	GmRBCS^{79}_pUC57 Kan	---	NcoI/KpnI	GmRBCS^{79}	AtHP59^{93}-BicA-mGFP6-6xHIS-MYC_pUC57 Amp	GmRBCS^{79}-BicA-mGFP6-6xHIS-MYC_pUC57 Amp	GmRBCS^{79}-BicA-mGFP6-6xHIS-MYC_pMDC32
	AtPLGG1^{92}-BicA	AtPLGG1^{92}-BicA_pUC57 Kan	---	NcoI/BamHI	AtPLGG1^{92}-BicA	AtHP59^{93}-BicA-mGFP6-6xHIS-MYC_pUC57 Amp	AtPLGG1^{92}-BicA-mGFP6-6xHIS-MYC_pUC57 Amp	AtPLGG1^{92}-BicA-mGFP6-6xHIS-MYC_pMDC32
	AtHP59^{93}-SbtA	AtHP59^{93}-SbtA_pUC57 Kan	---	NcoI/BamHI	AtHP59^{93}-SbtA	AtHP59^{93}-BicA-mGFP6-6xHIS-MYC_pUC57 Amp	AtHP59^{93}-SbtA-mGFP6-6xHIS-MYC_pUC57 Amp	AtHP59^{93}-SbtA-mGFP6-6xHIS-MYC_pMDC32
	AtPLGG1^{92}-SbtA	AtPLGG1^{92}-SbtA_pUC57 Kan	---	NcoI/BamHI	AtPLGG1^{92}-SbtA	AtHP59^{93}-BicA-mGFP6-6xHIS-MYC_pUC57 Amp	AtPLGG1^{92}-SbtA-mGFP6-6xHIS-MYC_pUC57 Amp	AtPLGG1^{92}-SbtA-mGFP6-6xHIS-MYC_pMDC32
	GmRBCS^{cTP}-BicA	GmRBCS^{cTP}-BicA_pUC57 Kan	---	NcoI/BamHI	GmRBCS^{cTP}-BicA	GmRBCS^{79}-BicA-mGFP6-6xHIS-MYC_pUC57 Amp	GmRBCS^{cTP}-BicA-mGFP6-6xHIS-MYC_pUC57 Amp	GmRBCS^{cTP}-BicA-mGFP6-6xHIS-MYC_pMDC32
	AtNTT1^{cTP}-BicA	AtNTT1^{cTP}-BicA_pUC57 Kan	---	NcoI/BamHI	AtNTT1^{cTP}-BicA	GmRBCS^{79}-BicA-mGFP6-6xHIS-MYC_pUC57 Amp	AtNTT1^{cTP}-BicA-mGFP6-6xHIS-MYC_pUC57 Amp	AtNTT1^{cTP}-BicA-mGFP6-6xHIS-MYC_pMDC32
	AtNTT1^{50}-BicA	AtNTT1^{50}-BicA_pUC57 Kan	---	NcoI/BamHI	AtNTT1^{50}-BicA	GmRBCS^{79}-BicA-mGFP6-6xHIS-MYC_pUC57 Amp	AtNTT1^{50}-BicA-mGFP6-6xHIS-MYC_pUC57 Amp	AtNTT1^{50}-BicA-mGFP6-6xHIS-MYC_pMDC32
	AtNTT1^{60}-BicA	AtNTT1^{60}-BicA_pUC57 Kan	---	NcoI/BamHI	AtNTT1^{60}-BicA	GmRBCS^{79}-BicA-mGFP6-6xHIS-MYC_pUC57 Amp	AtNTT1^{60}-BicA-mGFP6-6xHIS-MYC_pUC57 Amp	AtNTT1^{60}-BicA-mGFP6-6xHIS-MYC_pMDC32
	AtNTT1^{70}-BicA	AtNTT1^{70}-BicA_pUC57 Kan	---	NcoI/BamHI	AtNTT1^{70}-BicA	GmRBCS^{79}-BicA-mGFP6-6xHIS-MYC_pUC57 Amp	AtNTT1^{70}-BicA-mGFP6-6xHIS-MYC_pUC57 Amp	AtNTT1^{70}-BicA-mGFP6-6xHIS-MYC_pMDC32
	AtNTT1^{115}-BicA	AtNTT1^{115}-BicA_pUC57 Kan	---	NcoI/BamHI	AtNTT1^{115}-BicA	GmRBCS^{79}-BicA-mGFP6-6xHIS-MYC_pUC57 Amp	AtNTT1^{115}-BicA-mGFP6-6xHIS-MYC_pUC57 Amp	AtNTT1^{115}-BicA-mGFP6-6xHIS-MYC_pMDC32
	AtHP59$^{\Delta cTP}$	AtHP59$^{\Delta cTP}$_pUC57 Kan	---	NcoI/BamHI	AtHP59$^{\Delta cTP}$	GmRBCS^{79}-BicA-mGFP6-6xHIS-MYC_pUC57 Amp	AtHP59$^{\Delta cTP}$-mGFP6-6xHIS-MYC_pUC57 Amp	AtHP59$^{\Delta cTP}$-mGFP6-6xHIS-MYC_pMDC32
	AtPLGG1$^{\Delta cTP}$	AtPLGG1$^{\Delta cTP}$_pUC57 Kan	---	NcoI/BamHI	AtPLGG1$^{\Delta cTP}$	GmRBCS^{79}-BicA-mGFP6-6xHIS-MYC_pUC57 Amp	AtPLGG1$^{\Delta cTP}$-mGFP6-6xHIS-MYC_pUC57 Amp	AtPLGG1$^{\Delta cTP}$-mGFP6-6xHIS-MYC_pMDC32
	AtHP59$^{\Delta MPL}$	AtHP59$^{\Delta MPL}$_pUC57 Kan	---	NcoI/BamHI	AtHP59$^{\Delta MPL}$	GmRBCS^{79}-BicA-mGFP6-6xHIS-MYC_pUC57 Amp	AtHP59$^{\Delta MPL}$-mGFP6-6xHIS-MYC_pUC57 Amp	AtHP59$^{\Delta MPL}$-mGFP6-6xHIS-MYC_pMDC32
	AtPLGG1$^{\Delta MPL}$	AtPLGG1$^{\Delta MPL}$_pUC57 Kan	---	NcoI/BamHI	AtPLGG1$^{\Delta MPL}$	GmRBCS^{79}-BicA-mGFP6-6xHIS-MYC_pUC57 Amp	AtPLGG1$^{\Delta MPL}$-mGFP6-6xHIS-MYC_pUC57 Amp	AtPLGG1$^{\Delta MPL}$-mGFP6-6xHIS-MYC_pMDC32
	AtHP59^{invMPL}	AtHP59^{invMPL}_pUC57 Kan	---	NcoI/BamHI	AtHP59^{invMPL}	GmRBCS^{79}-BicA-mGFP6-6xHIS-MYC_pUC57 Amp	AtHP59^{invMPL}-mGFP6-6xHIS-MYC_pUC57 Amp	AtHP59^{invMPL}-mGFP6-6xHIS-MYC_pMDC32
	AtPLGG1^{invMPL}	AtPLGG1^{invMPL}_pUC57 Kan	---	NcoI/BamHI	AtPLGG1^{invMPL}	GmRBCS^{79}-BicA-mGFP6-6xHIS-MYC_pUC57 Amp	AtPLGG1^{invMPL}-mGFP6-6xHIS-MYC_pUC57 Amp	AtPLGG1^{invMPL}-mGFP6-6xHIS-MYC_pMDC32
	AtHP59$^{PLGG1(MPL)}$	AtHP59$^{PLGG1(MPL)}$_pUC57 Kan	---	NcoI/BamHI	AtHP59$^{PLGG1(MPL)}$	GmRBCS^{79}-BicA-mGFP6-6xHIS-MYC_pUC57 Amp	AtHP59$^{PLGG1(MPL)}$-mGFP6-6xHIS-MYC_pUC57 Amp	AtHP59$^{PLGG1(MPL)}$-mGFP6-6xHIS-MYC_pMDC32
	AtPLGG1$^{HP59(MPL)}$	AtPLGG1$^{HP59(MPL)}$_pUC57 Kan	---	NcoI/BamHI	AtPLGG1$^{HP59(MPL)}$	GmRBCS^{79}-BicA-mGFP6-6xHIS-MYC_pUC57 Amp	AtPLGG1$^{HP59(MPL)}$-mGFP6-6xHIS-MYC_pUC57 Amp	AtPLGG1$^{HP59(MPL)}$-mGFP6-6xHIS-MYC_pMDC32
	AtHP59^{cTP}-BicA	AtHP59^{cTP}-BicA_pUC57 Kan	---	NcoI/BamHI	AtHP59^{cTP}-BicA	GmRBCS^{79}-BicA-mGFP6-6xHIS-MYC_pUC57 Amp	AtHP59^{cTP}-BicA-mGFP6-6xHIS-MYC_pUC57 Amp	AtHP59^{cTP}-BicA-mGFP6-6xHIS-MYC_pMDC32
	AtPLGG1^{cTP}-BicA	AtPLGG1^{cTP}-BicA_pUC57 Kan	---	NcoI/BamHI	AtPLGG1^{cTP}-BicA	GmRBCS^{79}-BicA-mGFP6-6xHIS-MYC_pUC57 Amp	AtPLGG1^{cTP}-BicA-mGFP6-6xHIS-MYC_pUC57 Amp	AtPLGG1^{cTP}-BicA-mGFP6-6xHIS-MYC_pMDC32
	AtHP59^{145}-SbtA	AtHP59^{145}_pUC57 Kan	---	NcoI/KpnI	AtHP59^{145}	AtPLGG1^{92}-SbtA-mGFP6-6xHIS-MYC_pUC57 Amp	AtHP59^{145}-SbtA-mGFP6-6xHIS-MYC_pUC57 Amp	AtHP59^{145}-SbtA-mGFP6-6xHIS-MYC_pMDC32
	AtPLGG1^{121}-SbtA	AtPLGG1^{121}_pUC57 Kan	---	NcoI/KpnI	AtPLGG1^{121}	AtPLGG1^{92}-SbtA-mGFP6-6xHIS-MYC_pUC57 Amp	AtPLGG1^{121-} SbtA-mGFP6-6xHIS-MYC_pUC57 Amp	AtPLGG1^{121}-SbtA-mGFP6-6xHIS-MYC_pMDC32
	AtHP59^{cTP}-SbtA	AtPLGG1^{121-} SbtA-mGFP6-6xHIS-MYC_pUC57 Amp	---	KpnI/BamHI	SbtA	AtHP59^{cTP}-BicA-mGFP6-6xHIS-MYC_pUC57 Amp	AtHP59^{cTP}-SbtA-mGFP6-6xHIS-MYC_pUC57 Amp	AtHP59^{cTP}-SbtA-mGFP6-6xHIS-MYC_pMDC32
	AtPLGG1^{cTP}-SbtA	AtPLGG1^{121-} SbtA-mGFP6-6xHIS-MYC_pUC57 Amp	---	KpnI/BamHI	SbtA	AtPLGG1^{cTP}-BicA-mGFP6-6xHIS-MYC_pUC57 Amp	AtPLGG1^{cTP}-SbtA-mGFP6-6xHIS-MYC_pUC57 Amp	AtPLGG1^{cTP}-SbtA-mGFP6-6xHIS-MYC_pMDC32

Table S1. Cloning steps to generate the constructs used in this study. Construct names written in blue indicate synthesized constructs. Colored boxed highlight identical plasmids. Constructs are grouped per cloning strategy.

Primer name	Primer sequence
26	GGGGACAAGTTTGTACAAAAAAGCAGGCTTCATGGCTTTCGCTGTCTC
27	GGGGACCACTTTGTACAAGAAAGCTGGGTCTTATTCATTAAGATCCTCCTCAGA
28	GGGGACAAGTTTGTACAAAAAAGCAGGCTTCATGGCTACTCTTTTAGCCACTC
30	GGGGACAAGTTTGTACAAAAAAGCAGGCTTCATGGGCACGGAGGACTAC
31	GGGGACAAGTTTGTACAAAAAAGCAGGCTTCATGGGTAACAAGGACTATGAGTACC
52	GGGGACAAGTTTGTACAAAAAAGCAGGCTTCATGCAGATAACCAACAAAATTCAC
68	GGGGACAAGTTTGTACAAAAAAGCAGGCTTCATGGATTTCTTGTCCAATTTCTTAAT
98	GGGGACAAGTTTGTACAAAAAAGCAGGCTTCATGGAAGCTGTGATTCAAACC

Table S2. PCR primers used for cloning. Gene sequences, extra bases and attB sequences are highlighted in black, grey and red, respectively.

**Leader name**	**Protein sequence**
Gm RBCS^{cTP}	MASSMISSPAVTTVNRAGAGMVAPFTGLKSMAGFPTRKTNNDITSIASNGGRVQ
GmPs RBCS^{79}	MASSMISSPAVTTVNRAGAGMVAPFTGLKSMAGFPTRKTNNDITSIASNGGRVQCMQVWPPIGKKKFETLSYLPPLTRD
Ps RBCS^{cTP}	MASMISSSAVTTVSRASRGQSAAVAPFGGLKSMTGFPVKKVNTDITSITSNGGRVK
Gm RBCS^{79}	MASSMISSPAVTTVNRAGAGMVAPFTGLKSMAGFPTRKTNNDITSIASNGGRVQCMQVWPPVGKKKFETLSYLPDLDDA
*At*NTT1^{cTP}	MEAVIQTRGLLSLPTKPIGVR
*At*NTT1^{50}	MEAVIQTRGLLSLPTKPIGVRSQLQPSHGLKQRLFAAKPRNLHGLSLSFN
*At*NTT1^{60}	MEAVIQTRGLLSLPTKPIGVRSQLQPSHGLKQRLFAAKPRNLHGLSLSFNGHKKFQTFEP
*At*NTT1^{70}	MEAVIQTRGLLSLPTKPIGVRSQLQPSHGLKQRLFAAKPRNLHGLSLSFNGHKKFQTFEPTLHGISISHK
*At*NTT1^{115}	MEAVIQTRGLLSLPTKPIGVRSQLQPSHGLKQRLFAAKPRNLHGLSLSFNGHKKFQTFEPTLHGISISHKERSTEFICKAEAAA<br>AGDGAVFGEGDSAAVVASPKIFGVEVATLKK
*At*HP59^{cTP}	MAFAVSVQSHFAIRALKRDHFKNPSPRTFCS
*At*HP59^{93}	MAFAVSVQSHFAIRALKRDHFKNPSPRTFCSCFKSRPDSSYLSLKERTCFVSKPGLVTTRYRHIFQVGAETGGEFADSGEVAD<br>SLASDAPESF
*At*HP59^{145}	MAFAVSVQSHFAIRALKRDHFKNPSPRTFCSCFKSRPDSSYLSLKERTCFVSKPGLVTTRYRHIFQVGAETGGEFADSGEVAD<br>SLASDAPESFSWSSVILPFIFPALGGLLFGYDIGATSGATLSLQSPALSGTTWFNFSPVQLG
*At*PLGG1^{cTP}	MATLLATPIFSPL
*At*PLGG1^{92}	MATLLATPIFSPLASSPARNRLSCSKIRFGSKNGKILNSDGAQKLNLSKFRKPDGQRFLQMGSSKEMNFERKLSVQAMDGAG<br>TGNTSTISRN
*At*PLGG1^{121}	MATLLATPIFSPLASSPARNRLSCSKIRFGSKNGKILNSDGAQKLNLSKFRKPDGQRFLQMGSSKEMNFERKLSVQAMDGAG<br>TGNTSTISRNVIAISHLLVSLGIILAADYFLKQAFVAAS

Table S3. Amino acid sequence of all leaders presented in this study and not included in previous figures.
